# Use of temporal contact graphs to understand the evolution of COVID-19 through contact tracing data

**DOI:** 10.1038/s42005-022-01045-4

**Published:** 2022-11-04

**Authors:** Mincheng Wu, Chao Li, Zhangchong Shen, Shibo He, Lingling Tang, Jie Zheng, Yi Fang, Kehan Li, Yanggang Cheng, Zhiguo Shi, Guoping Sheng, Yu Liu, Jinxing Zhu, Xinjiang Ye, Jinlai Chen, Wenrong Chen, Lanjuan Li, Youxian Sun, Jiming Chen

**Affiliations:** 1 State Key Laboratory of Industrial Control Technology, Zhejiang University, Hangzhou, 310027 China; 2 College of Control Science and Engineering, Zhejiang University, Hangzhou, 310027 China; 3 Shulan (Hangzhou) Hospital Affiliated to Shulan International Medical College, Zhejiang Shuren University, Hangzhou, 310015 China; 4 Zhejiang Institute of Medical-care Information Technology, Hangzhou, 311100 China; 5 Westlake Institute for Data Intelligence, Hangzhou, 310012 China; 6 College of Information Science and Electronic Engineering, Zhejiang University, Hangzhou, 310027 China; 7 State Key Laboratory for Diagnosis and Treatment of Infectious Diseases, Zhejiang University, Hangzhou, 310027 China

**Keywords:** Computational science, Statistics

## Abstract

Digital contact tracing has been recently advocated by China and many countries as part of digital prevention measures on COVID-19. Controversies have been raised about their effectiveness in practice as it remains open how they can be fully utilized to control COVID-19. In this article, we show that an abundance of information can be extracted from digital contact tracing for COVID-19 prevention and control. Specifically, we construct a temporal contact graph that quantifies the daily contacts between infectious and susceptible individuals by exploiting a large volume of location-related data contributed by 10,527,737 smartphone users in Wuhan, China. The temporal contact graph reveals five time-varying indicators can accurately capture actual contact trends at population level, demonstrating that travel restrictions (e.g., city lockdown) in Wuhan played an important role in containing COVID-19. We reveal a strong correlation between the contacts level and the epidemic size, and estimate several significant epidemiological parameters (e.g., serial interval). We also show that user participation rate exerts higher influence on situation evaluation than user upload rate does, indicating a sub-sampled dataset would be as good at prediction. At individual level, however, the temporal contact graph plays a limited role, since the behavior distinction between the infected and uninfected individuals are not substantial. The revealed results can tell the effectiveness of digital contact tracing against COVID-19, providing guidelines for governments to implement interventions using information technology.

## Abstract (web summary)

Contact tracing using mobile phones has been a strategy used by many countries to limit the spread of COVID-19. Here, the authors use location-related phone data from Wuhan to construct a temporal contact graph and demonstrate a strong correlation between contacts level and epidemic size, thus justifying the effectiveness of the approach.

## Introduction

COVID-19, caused by SARS-CoV-2, has rapidly spread to most of the countries in the past year. As of 11 AM CEST, 8 September 2022, world-wide confirmed cases of COVID-19 has reached 603,711,760, among which 6,484,136 patients died^1^. It has been overwhelming the medical systems of many countries with large case counts and threatening to infect an extremely large population, but it is still too early to tell its disappearance^2^. Currently, many countries (e.g., the U.S.A., the U.K., Australia, etc.) have been cooperating together to prevent and control such an unprecedented disease via a variety of ways^3–5^.

As is known, contact tracing is one of the most effective ways to search individuals with a high risk of being infected. However, it is always costly, time-consuming, and even impossible to find unconscious contacts by traditional ways. Recently, digital contact tracing using information technology has been widely advocated to replace traditional labor-intensive contact surveys^5–7^. The main idea is to exploit Bluetooth/positioning sensors on smartphones to discover nearby devices held by users and identify the contacts with the infectious individuals^8,9^. On one hand, about 28 countries such as China, Switzerland, Spain, the United Kingdom, Australia, Singapore and Germany have implemented various measures using information technology (e.g., launching digital contact tracing apps)^10–14^. On the other hand, however, recent works have revealed that digital contact tracing contributes little to contain outbreaks, principally because of low participation rates and low engagement of participants^15,16^. As many controversial issues of digital contact tracing have been raised, it is urgent to review empirical evidence for the effectiveness of this measure against a pandemic spreading from different aspects^17–19^.

Since contact tracing measures are essentially based on crowdsourcing^20^, their performance highly relies on the involvement of voluntary smartphone users. Due to potential privacy leakage and cost incurred during crowdsourcing process, voluntary users are reluctant to participate and contribute their personal data at a fine-grained scale^21^. It is challenging to fully utilize sparse and noisy crowdsourced data of contact information from voluntary users to capture the intrinsic transmission characteristic of COVID-19. Therefore, we devote ourselves to take an in-depth investigation into this issue, and to show that an abundance of information can be extracted from digital contact tracing for COVID-19 prevention and control. This is different from previous studies which focused on integrating mathematical models and available statistical data of confirmed cases to characterize the transmission of epidemic diseases^22–38^, or those which utilized individual mobility traces (with the information of confirmed cases) to simulate the spreading process^7,39^, providing insight to understand the spreading of COVID-19 from the aspect of digital contact tracing.

**Fig. 1:**
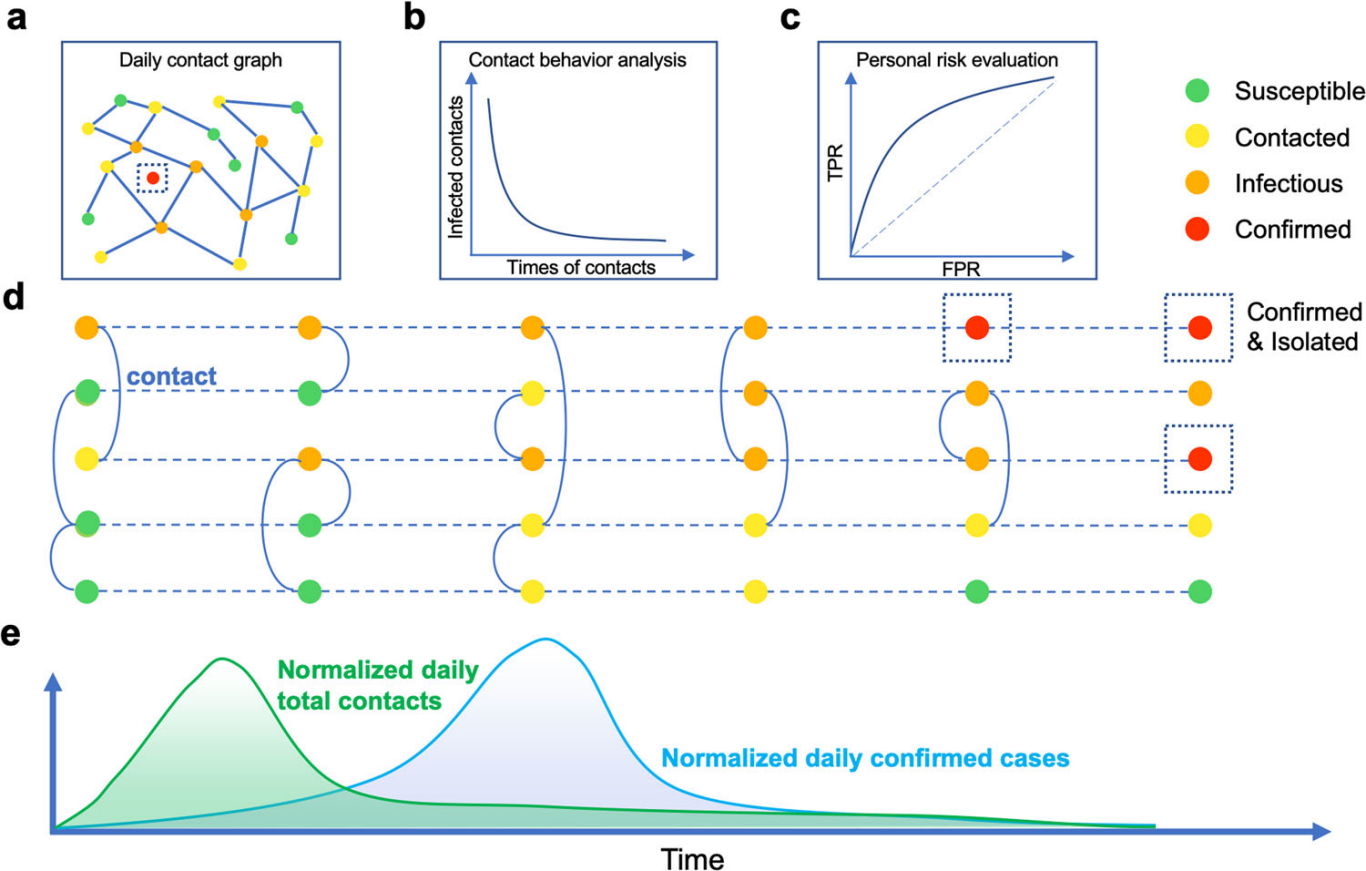
Temporal contact graph and schematics for its potential applications. An individual has four status: susceptible, contacted, infectious and confirmed. The status `susceptible' turns to `contacted' when an individual had at least one contact with infectious individuals. A contacted individual may be infected or stay healthy. The status `infectious' changes to `confirmed' when confirmation is made. In China, confirmed cases will be quarantined for treatment and no longer infectious to others. **a** Daily contact graph. **b** The analysis for contact behaviors shows the distributions of contact counts between infected and uninfected contacted individuals. **c** The personal risk evaluation based on contact behaviors. **d** Contact history and status of individuals. A node denotes an individual and different colors indicate different status. A dashed line means the status evolution of a single individual in timeline, and a solid curve between two individuals means a contact. **e** The correlation between normalized daily total contacts and daily confirmed cases. The panels here are illustrative examples.

In this article, we construct a temporal contact graph (Fig. 1a, d) that quantifies the daily contacts between infectious and susceptible individuals by exploiting a large volume of location-related data contributed by a large volume of smartphone users in Wuhan, China. We demonstrate that such a temporal contact graph has many applications, e.g., to analyze the dynamic contact behavior (Fig. 1b), identify the potential infected contacted individuals (Fig. 1c), and assist the decision-making of control measures (Fig. 1e). Specifically, we use five time-varying indicators that are validated to have the capability of accurately capturing actual contact trends at individual and population level in Wuhan, providing a data-driven evidence that the travel restrictions in Wuhan significantly reduced the chance of susceptible individuals having contacts with the infectious and thus played an important role in containing COVID-19. We reveal a strong correlation between the number of daily symptomatic cases and daily total contacts with a 12-days delay, and estimate several significant epidemiological parameters such as the serial interval. We study the effect of user involvement on the effectiveness of digital contact tracing measures, finding that user participation rate exerts higher influence on situation evaluation than user upload rate does. By dividing all individuals into two groups, i.e., the infected and the uninfected, we show that the contact distinction of the two groups are not significant. Moreover, the contact distinction is more significant than the gender distinction but less significant than the age distinction. By designing an infection risk evaluation framework, we find it only performs a limited role in identifying high-risk contacted individuals. This indicates that it is not highly effective to narrow down the search of high risk contacted individuals for quarantine by the distinction of contact behaviors. The empirical results can offer a promising way to evaluate and predict the evolving epidemic situation of COVID-19, and provide guidelines for governments to implement digital contact tracing measures.

## Results

### Characteristics of informative indicators

We leverage a large volume of location-related data set contributed by 10,527,737 smartphone users in Wuhan, China. Each item in the data set includes a geohash encoded meshed area, a timestamp and an anonymized identity. We build a contact model, in which a contact between two individuals is said to occur when they are reported within a temporal interval of 120 min, and a certain spatial area of 15 m × 29 m according to the uploaded geohash. By collaborating with local authority, we obtain the information whether and when each anonymous individual was confirmed. With the information of 14,198 confirmed cases and the contact model we build, we identify 519,400 contacted individuals, with which we are able to construct a temporal contact graph (consisting of over 2.4 million contacts) between infectious and susceptible individuals. We use five informative indicators (*t* denotes a day): (1) *C*(*t*), the daily total number of contacts between infectious and contacted individuals, i.e. the number of edges in the constructed temporal contact graph; (2) *I*(*t*), the daily number of infectious individuals who had encountered with contacted individuals at least once, i.e. the number of infectious nodes; (3) *S*(*t*), the daily number of contacted individuals who had encountered the infectious at least once, i.e. the number of susceptible nodes; (4) *k*_*I*_(*t*), the daily average contacts of infectious individuals associating with contacted individuals, i.e., the average degree of infectious nodes, and (5) *k*_*S*_(*t*), the daily average contacts of contacted individuals associating with infectious individuals, i.e., the average degree of susceptible nodes.

**Fig. 2:**
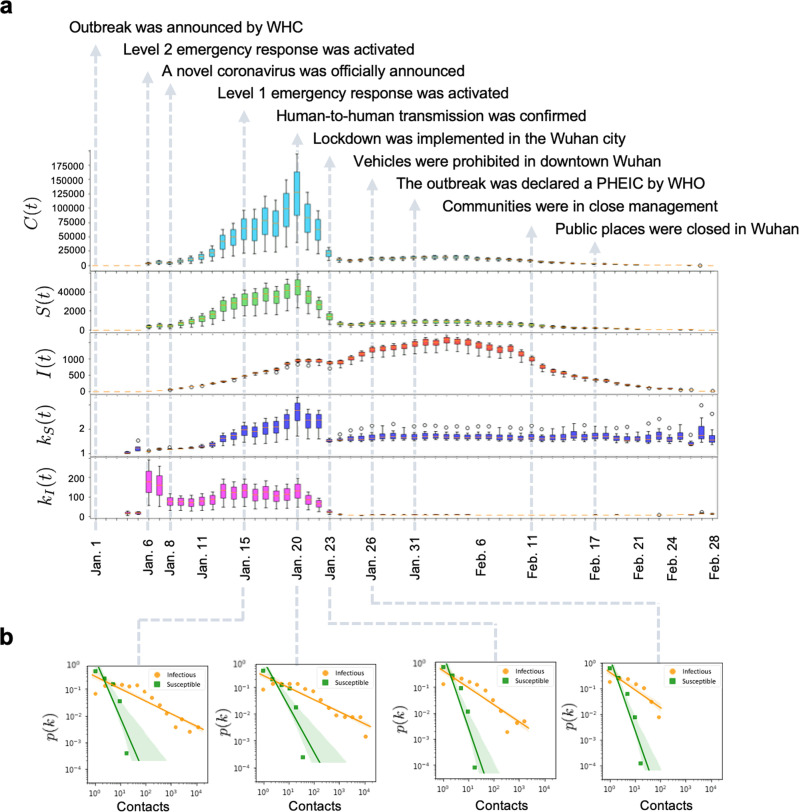
Daily characteristics of five indicators. **a** *C*(*t*), the daily total number of contacts between infectious and contacted individuals. *S*(*t*), the daily total number of contacted individuals who had encountered the infectious at least once. *I*(*t*), the daily total number of infectious individuals who had encountered with contacted individuals at least once. *k*_*S*_(*t*), the daily average number of infectious individuals that each susceptible individual encountered. *k*_*I*_(*t*), the daily average number of susceptible individuals that each infectious individual contacted. The error bar in this panel indicates the standard deviation of different time interval *T* in the contact model, which varies from 15 to 120 min. **b** The distributions *p*(*k*) of the daily number (*k*) of contacts by all contacted individuals and the distributions *p*(*k*) of the daily number (*k*) of contacts by all confirmed cases, respectively, on four specific days.

The five indicators at the beginning of 2020 are shown along with a series of implements (Fig. 2a). The daily total contacts between infectious and susceptible individuals *C*(*t*) can reflect the potential transmission. We find that *C*(*t*) increased dramatically first from 4 to 20 January 2020, due to the fast increasing infectious individuals, and then dropped after 20 January. As we know, the Chinese authority announced the outbreak of COVID-19 and confirmed its infection among people on 20 January, which explains the decline of *C*(*t*). Obviously, *C*(*t*) decreased sharply around 23 January when the lockdown was implemented in Wuhan, and tended to zero around 28 February.

From a macroscopic view, *S*(*t*) and *I*(*t*) describe population-level contacts trend in Wuhan. Notice that *S*(*t*) had a minor bouncing back after 26 January 2020, which is possibly due to the number of confirmed cases quickly increased after 23 January, and people in Wuhan could still move within the city (their mobility increased due to the approaching of Chinese New Year). Then, *S*(*t*) began to decline on 4 February, and approached zero around 28 February. Compared to *S*(*t*), however, *I*(*t*) performs a different characteristic from the other ones. Initially, *I*(*t*) quickly increased with the number of confirmed cases as few of them are under quarantine. It began to drop on 20 January upon the official announcement and reached the local minimum on 23 January, after which it had a duration of increase. It decreased again on 3 February and eventually approached to zero around 28 February. The main reason is that the confirmed cases increased fast after 20 January and the chance of meeting an infectious individual remained high as many of them were not hospitalized due to test capacity constraint.

Further evidence can be observed from the indicators *k*_*S*_(*t*) and *k*_*I*_(*t*). *k*_*S*_(*t*) performed a similar behavior as *S*(*t*), while *k*_*I*_(*t*) displays a more distinct fluctuation in the early January 2020, since the infected are not isolated, and they contacted the susceptible as usual in the incubation period. On account of the small proportion of the infected and the randomness of their movements, the two indicators were not stable during 6–20 January. For example, they first dropped a bit around 10 January, which may be due to the mobility reduction caused by the sudden drop of temperature (Supplementary Fig. 2). The dynamic *k*_*S*_(*t*) and *k*_*I*_(*t*) accurately describe the actual individual-level contacts trend in Wuhan, providing data-driven evidence that travel restrictions in Wuhan significantly reduced the chance of a susceptible individual having contacts with the infectious individuals and thus played an important role in containing COVID-19.

From the perspective of the infectious, the distribution of daily contacts is heavy-tailed (Fig. 2b), and has a prominent long tail especially when the exponent coefficient is small before 23 January. The long tails indicate that there were some super active cases who had contacted with hundreds of susceptible individuals. Identifying and quarantining them helps mitigate the fast transmission. Therefore, *C*(*t*), *S*(*t*), *I*(*t*), *k*_*S*_(*t*), and *k*_*I*_(*t*), characterized the spread of COVID-19 from dimensions of susceptible individuals, confirmed cases and overall contacts, which were informative for COVID-19 prevention and control (see Supplementary Fig. 5 for more sensitivity analyses).

### A strong situation correlation revealed by digital contact tracing

The temporal contact graph shows the potential group of contacted individuals at high infection risk. Intuitively, more contacts between infectious and contacted individuals are likely to cause more confirmed cases in the future. We proceed to investigate the correlation between the daily number of contacts *C*(*t*) and the symptomatic cases reported by authority^40^.

**Fig. 3:**
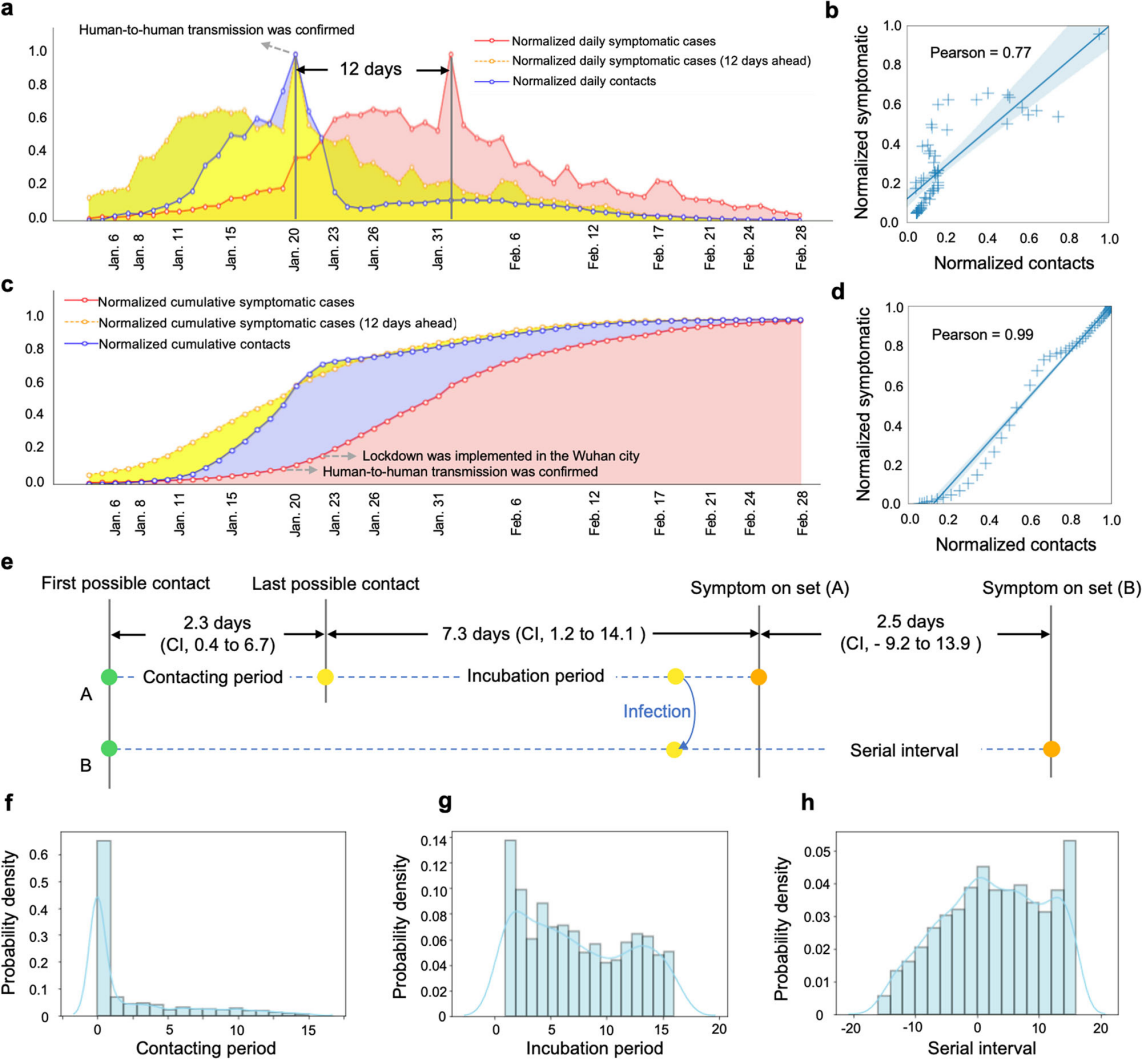
Daily and cumulative correlation analysis. **a** Historical time series of the number of daily contacts (in blue), daily reported symptomatic cases in Wuhan (in red) and daily reported symptomatic cases ahead 12 days (in yellow). **b** The reached maximum Pearson correlation (0.77) between normalized daily contacts and normalized daily confirmed cases with a 12-days delay. **c** Historical time series of the number of cumulative contacts (in blue), cumulative symptomatic cases in Wuhan (in red) and cumulative reported symptomatic cases with a 12-days delay (in yellow), where the Pearson correlation reaches 0.99. **d** The reached Pearson correlation (0.99) between normalized cumulative contacts and normalized cumulative symptomatic cases with a 12-days delay. **e** The timeline displays the contact period, incubation period, and serial interval inferred by digital contact measure. **f** The distribution of the duration from the first possible contact to the last possible contact (mean 2.3 days, 95% CI, 0.4 to 6.7 days). **g** The distribution of the duration from the last possible contact to symptom onset (mean 7.3 days, 95% CI, 1.2 to 14.1 days). **h** The distribution of the duration from the symptom onset of A to the symptom onset if B who is infected by A (mean 2.5 days, 95% CI, −9.2 to 13.9 days).

The curves of daily number of contacts *C*(*t*) (in blue) and daily symptomatic cases (in red) with normalization (i.e., normalized by the maximum) in Wuhan are shown in Fig. 3a, from which we observe a prominent delay between them. By moving points in the time series of daily number of total symptomatic cases ahead (in yellow), these two curves present more similar trends. To find the proper delay that results in the best similarity in trends between the curves of daily number of contacts and confirmed cases, we alter the delays ranging from 0 to 17 days according to existing surveys^41,42^. The experiments show that a 12-days delay results in the best Pearson correlation of 0.77 (Fig. 3b) in accordance with recent works^42–49^. As for the cumulative correlation analysis, the curves of cumulative contacts (in blue) and cumulative symptomatic cases (in red) with normalization (i.e., normalized by the maximum) in Wuhan are shown in Fig. 3c, where we find a strong correlation between the number of cumulative contacts and the cumulative confirmed cases with 12 days ahead (in yellow). The delay from being contacted to symptom onset may vary for different individuals, while analyzing the cumulative correlation would weaken these variations, reaching a higher Pearson correlation. Specifically, the Pearson correlation reaches 0.99 when there is a 12-days delay between normalized cumulative contacts and normalized cumulative symptomatic cases (Fig. 3d). Since the correlation between cumulative contacts and cumulative symptomatic cases is higher than that between daily contacts and daily symptomatic cases. Thus, the number of cumulative contacts can reflect and estimate the number of symptomatic cases with higher accuracy, having a better predictability of the number of symptomatic cases than the number of daily contacts does. In summary, indicator *C*(*t*) provides an empirical way to evaluate and predict the epidemic situation of COVID-19.

Furthermore, we also explore several significant epidemiology parameters including the contacting period, incubation period, and serial interval (Fig. 3e). Specifically, the contacting period indicates the interval from the first possible contact to the last possible contact, which is estimated to be 2.3 days (95% CI, 0.4 to 6.7 days) (Fig. 3f). The incubation period indicates the interval from the last possible contact to symptom onset, which is estimated to be 7.3 days (95% CI, 1.2 to 14.1 days) (Fig. 3g). The serial interval indicates the interval from symptom onset of A to symptom onset of B who is infected by A, which is a proxy of generation period from the infection of A to the infection of B who is infected by A. Notice that the serial interval could be negative because of asymptomatic transmissions, and it is estimated to be 2.5 days (95% CI, −9.2 to 13.9 days) (Fig. 3h). These estimations are in accordance with most existing survey^42–49^, demonstrating the effectiveness of revealing epidemic situation at population level by digital contact tracing.

### The impacts of user involvement on the contact tracing performance

Clearly, digital contact tracing is based on crowdsouring. Individual smartphone users are voluntary to participate in the process and upload their contact information. It remains open to tell how the performance of contact tracing (e.g., estimating *k*_*S*_(*t*) and *k*_*I*_(*t*) and daily confirmed cases) is affected by user involvement, raising the question on whether contact tracing measures can really work in practice. We study on this issue by taking into account two types of user involvement: user participation rate (the proportion of users in the whole population) and data uploading rate (their data reporting frequency per day). To simulate user involvement, we randomly choose *α*% users as the voluntary users, and *α*% data items each participating user uploading per day, and evaluate the corresponding performance loss.

**Fig. 4:**
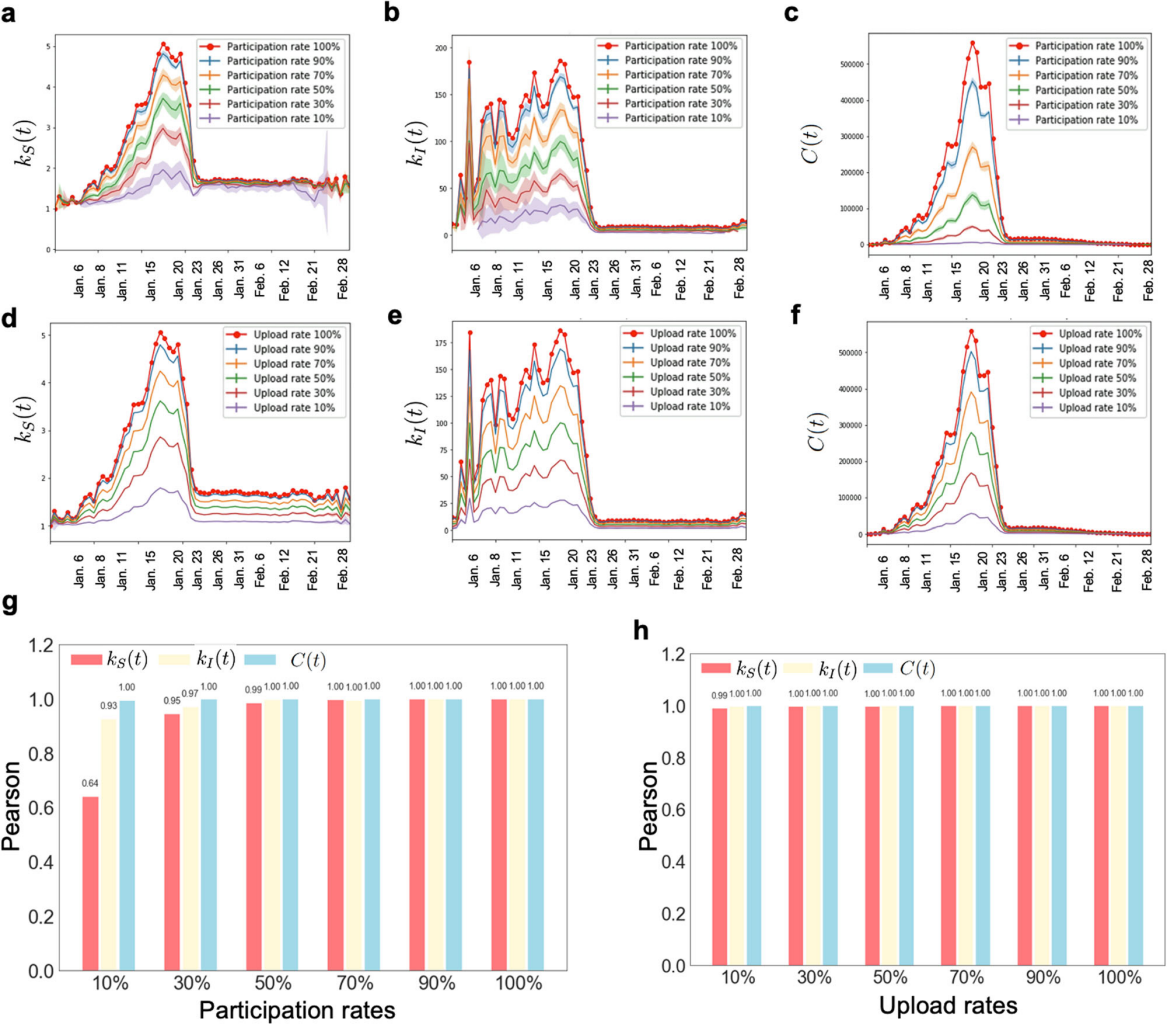
The performance of contact tracing under different user involvements. **a**–**c** Three figures show the change of the average degree of susceptible nodes *k*_*S*_(*t*), the average degree of infectious nodes *k*_*I*_(*t*), and the number of edges *C*(*t*) from 1 January to 28 February with error bars vs. different user participation rates. The error bar indicates the standard deviation of a ten-times repeating experiment. **d**–**f** Three figures show the change of daily *k*_*S*_(*t*), *k*_*I*_(*t*), and *C*(*t*) from 1 January to 28 February with error bars vs. different user upload rates. The error bar indicates the standard deviation of a ten-times repeating experiment. **g**, **h** The Pearson correlations vs. different user participation rates and user upload rates.

We conduct extensive explorations by varying the values of *α*, and repeat ten times of Monte Carlo experiments at each involvement level to make our experiments more credible. At a specific *α*, we plot the time series with error bars of *k*_*S*_(*t*), *k*_*I*_(*t*) and total contacts *C*(*t*) for both scenarios of user participation rate and user upload rate, ranging from 1 January to 28 February 2020. It is shown that, as *α* decreases, corresponding time series decrease with the similar trend (Fig. 4a–f). This is expected as reduction in either user participation rate or user upload rate decreases the chances of having contacts among users. To see if the reduction has influence on capturing the evolving trends, we calculate the Pearson correlations between the time series under *α*% and full (100%) participation rate/data upload rate case (Fig. 4g, h).

We obtain the following observations. (1) Decreasing the user upload rate or participation rate results in the lower values of *k*_*S*_(*t*), *k*_*I*_(*t*), and *C*(*t*). (2) User participation rate and data upload rate have minor effects on the evaluation of evolving pattern of *C*(*t*), whose error bars are not as obvious as another two variables. The above observations indicate that *C*(*t*) is more robust than *k*_*S*_(*t*) and *k*_*I*_(*t*) when user involvement changes. (3) *k*_*S*_(*t*) is more sensitive to the change of user involvement *α* than *k*_*I*_(*t*). This is because the number of susceptible individuals is much larger than that of the infectious. (4) User participation rate exerts higher influence on the three indicators than user upload rate does according to Fig. 4g, h. Therefore, we should encourage more user participation to obtain a better performance in practice. Considering their privacy and cost concerns, it would be a good strategy to allow voluntary smartphone users having a relatively low data upload rate. (5) For the participation rates analysis, when the participation rate reduces to 10%, the correlation coefficient reduces significantly according to Fig. 4g, which can be attributed to the characteristics of the overall heavy-tailed degree distribution of the network. Only when the participation rate is low enough can some key nodes be deleted, thereby affecting the trend of the entire network. The result indicates that it requires far less invasive data collection and a dramatically sub-sampled dataset would be as good at prediction, avoiding large-scale data collection. We note that the performance of individual-level infection risk evaluation will be impacted when user participation rate or upload rate drops since we may miss many contacts with infectious cases in such case and make an incorrect evaluation.

### Individual-level infection risk evaluation by contact behavior discrimination

In a spreading process, contacted individuals have chance of being infected, or staying healthy. We proceed to study the contact behaviors between the infected and uninfected contacted individuals, based on which we can obtain an individual-level infection risk evaluation. We count the number of contacts each contacted individual had with the infectious in recent 17 days, i.e., the infectious period (see Supplementary Note II for more sensitivity analysis), and calculate the probability *p*(*k*) that a contacted individual had *k* contacts for infected and uninfected contacted individuals, respectively.

**Fig. 5:**
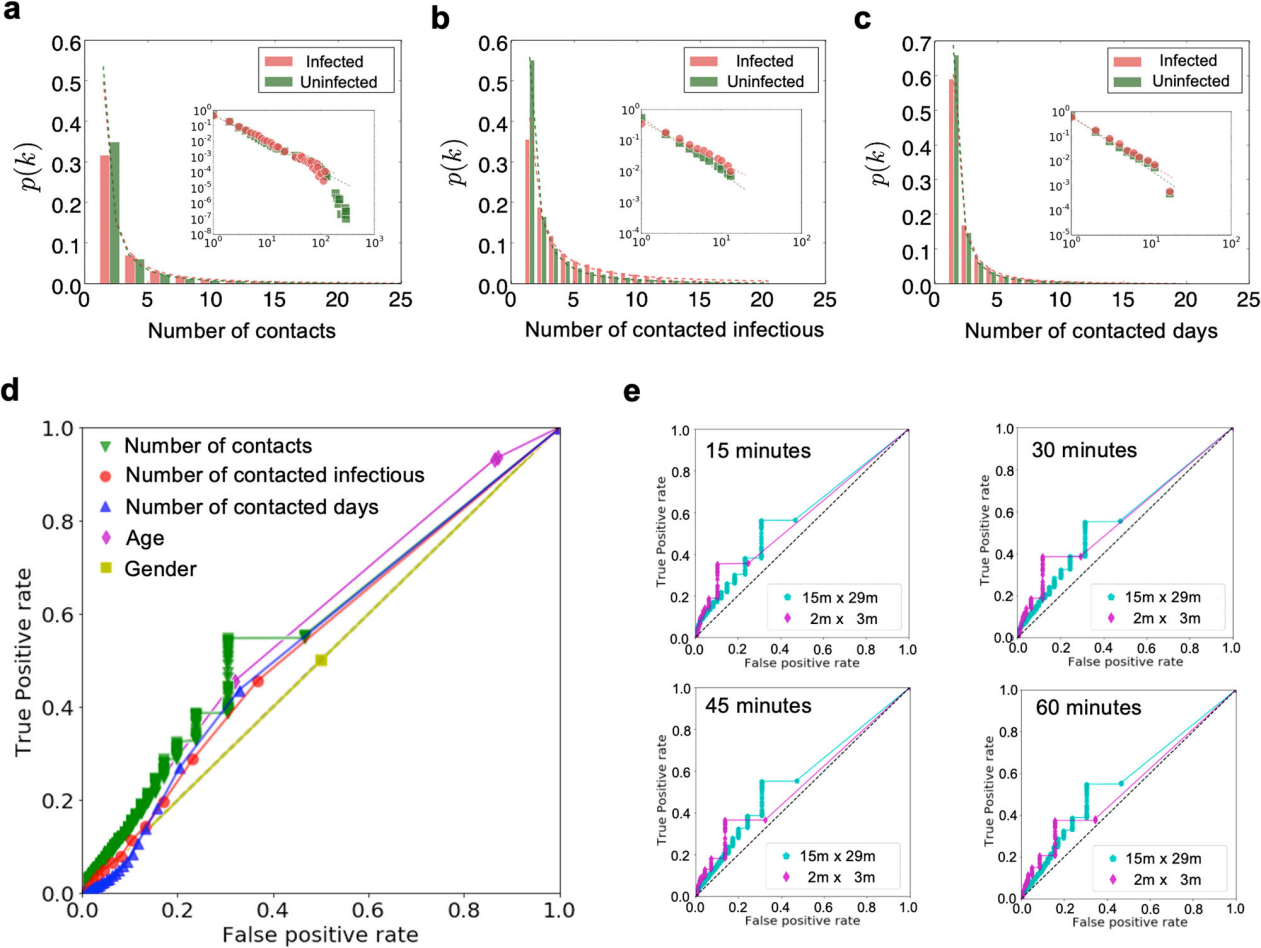
Infection risk evaluation based on the Bayesian framework. **a** The distributions of the numbers of contacts with the infectious by infected and uninfected contacted individuals, respectively. **b** The distributions of the numbers of the infectious by infected and uninfected contacted individuals, respectively. **c** The distributions of the days of contacts with the infectious by infected and uninfected contacted individuals, respectively. **d** The ROC curves for the risk evaluation. Here the *x*-axis denotes the false positive rate and the *y*-axis denotes the true positive rate, where a random guess gives a point along the dashed diagonal line. **e** The ROC (receiver operating characteristic) curves for the risk evaluation with different temporal and spatial granularities.

Heavy-tailed distributions are found for both types of the behaviors, while the parameters are mildly different if we fit them by power-law distributions. The contacts of infected contacted individuals can be fitted by a power-law distribution with an average <*k*>= 5.93 and an exponent *γ* = 1.66, while the contacts of uninfected contacted individuals can be fitted by a power-law distribution with <*k*> = 5.38 and *γ* = 1.81 (Fig. 5a).

Further, we count the number of infectious individuals who had contacts with any contacted individual in recent 17 days, and calculate the probability *p*(*k*) that a contacted individual have associated *k* infectious individuals for infected and uninfected contacted individuals, respectively. The infected contacted individuals have a fitted power-law distribution with <*k*> = 3.95 and *γ* = 1.33, and the uninfected contacted individuals have a fitted power-law distribution with <*k*> = 2.89 and *γ* = 1.79 (Fig. 5b). We count the number of days when contacted individuals had contacts with any infectious individual. The probability *p*(*k*) that a contacted individual have encountered any infectious individual for *k* days in recent 17 day for infected and uninfected contacted individuals, respectively. It can be fitted by a power-law distribution with <*k*> = 2.27 and an exponent *γ* = 1.94 for the infected contacted individuals, while it can be fitted by a fitted power-law distribution with <*k*> = 2.03 and *γ* = 2.22 for the uninfected contacted individuals (Fig. 5c). These distributions are different in terms of the expectations and the fitted power exponents: the infected contacted individuals have more contacts than uninfected contacted individuals and the corresponding distribution has a fatter tail. This indicates that there are an appreciable quantity of infected contacted individuals with a large amount of contacts.

Based on these contact behavior discriminations, we proceed to perform an individual-level infection risk evaluation for each contacted individual. We propose a risk evaluation method based on the Bayesian framework by calculating the posterior infected probability for any contacted individual^50^. We first use a variable *z*_*j*_ to represent the health status for any contacted individual *j*, i.e., *z*_*j*_ = 1 if *j* is infected and *z*_*j*_ = 0 otherwise. Then, the infection risk for *j* is determined by the posterior probability *P*(*z*_*j*_ = 1∣*b*_*j*_, *f*_*j*_):

1 $$P({z}_{j}=1| {b}_{j},{f}_{j})=\frac{P({b}_{j},{f}_{j}| {z}_{j}=1)\cdot P({z}_{j}=1)}{P({b}_{j},{f}_{j})},$$

where *b*_*j*_ denotes the contact features (e.g., number of contacts) for *j*, and *f*_*j*_ denotes the individual feature, indicating the age, gender, and etc. The term *P*(*b*_*j*_, *f*_*j*_∣*z*_*j*_ = 1) is the likelihood, and *P*(*z*_*j*_ = 1) indicates the infected probability for any contacted individual *j* a prior, which is taken as a constant (see the “Methods” section for more details).

After calculating the infection risk of every contacted individual, we vary the positive threshold from 0 to 1 and display the ROC (receiver operating characteristic) curve. The ROC space is defined by plotting the false positive rate in *x*-axis and the true positive rate as *y*-axis, indicating the relative trade-offs between false positive (costs) and true positive (benefits) (Fig. 5d). Increasing the threshold results in fewer true positives and false positives. However, the true positive is larger than false positives, indicating the infection risk model is effective. Above an appropriate threshold, for example, we can find about 50% of the infected contacted individuals with 30% false report of the uninfected contacted individuals, where the AUC (area under the ROC curve) reaches 0.57 by using the contact graph (green line). The feature of gender did not contain any information to distinguish infected ones, where the AUC is 0.5, while the AUC with the feature of age reaches 0.59. Generally, a high AUC can help narrow down high risk contacted individuals for quarantine in practice. Obviously, information of the age provides a more accurate discrimination to identify the infected contacted individuals, while there is nearly no distinction by gender. The results indicate that the distinction of contact behaviors between the infected and uninfected contacted individuals are not prominent. Moreover, the contact distinction is more significant than the gender distinction but less significant than the age distinction. To perform a sensitivity analysis for the temporal and spatial granularities, we vary the time interval and spatial area in the contact model. Specifically, the time interval is ranging from 15 to 60 min, and the contact distance is ranging from an area of 2 m × 3 m to 15 m × 29 m. The ROC curves shows the parameters are not sensitive, indicating a stable analytical result (Fig. 5e).

## Conclusion

Since the emergence of COVID-19, researchers have proposed many mathematical models to characterize the transmission of COVID-19^22–25,51^. As digital contact tracing has been advocated by many countries, it rises the pressing issue of how to fully utilize such a new approach to contain COVID-19. Here, we provide the first collection of results that accurately characterize the evolving epidemic situation of COVID-19 by exploiting the temporal contact graph. Our approach offers a data-driven approach to evaluate and predict the evolving epidemic situation of COVID-19. Clearly, our data-driven approach and the traditional model-based approaches are complementary to characterize the transmission of COVID-19.

As the contact tracing data are still unavailable, their performance on COVID-19 prevention and control can not be directly evaluated. Some excellent studies have utilized large-scale smartphone data to capture mobility patterns^52^, and simulated the infection process due to the unavailability of user infection status^2,7^. Here, we leverage a large amount of location-related data contributed by a large volume of voluntary users to study such an issue. As we know the health status of smartphone users, we construct a temporal contact graph between susceptible and infectious individuals, which can be directly used to characterize the transmission of COVID-19. This distinguishes our work from most of the previous studies. We show that we can obtain a good performance in estimating and evaluating the epidemic situation even when user participation rate and data upload rate are low. We also demonstrate that user participation rate has a bigger impact than data upload rate on the estimations of the proposed indicators. Our results can provide guidelines for governments to practically deploy digital contact tracing measures.

## Methods

### Data collection and contact model

The data are contributed by 10,527,737 voluntary users in Wuhan, China, and collected by crowdsourcing platforms from our industry partners. The location-related information was authorized and uploaded every time smartphone users are using location-based services (see Supplementary Note I for more data descriptions). Privacy protection mechanisms such as perturbation and pseudonymization are adopted during data collection. The location-related information, including POI, GPS, geomagnetic, etc., is projected into meshed area. The confirmed cases from 20 January to 28 February 2020, serve as the sources of the infection. They are linked to the status of smartphone users by their phone number, which is validated by the local authorities.

Note that all individual location-related data and health status information were collected, stored and used by following the Personal Information Security Specification (2019) and Public Health Emergencies Regulations of China. All raw data was stored in specialized data servers with limited access by LBS providers. This article only utilizes the temporal contact graph that is derived from the raw data.

We propose a contact model based on the crowdsourced dataset: a contact between two smartphone users is said to occur when they report the identical geohash within a given time interval. As aforementioned, the geohash can be projected into a mesh area of a certain meshed area (e.g., 15 m × 29 m). This means that a contact is characterized when the distance between two smartphone users is within 18 m averagely. Such a definition is similar to that adopted by most contact tracing apps which exploit Bluetooth or GPS to decide a contact when two users are in a short distance. As smartphone users report data in a very low and irregular frequency, the contributed data are typically sparse. We would miss many contacts if we only count those where two smartphone users are reporting identical information simultaneously. Considering the data sparsity, we define a contact occurring when two users upload the same geohash with time interval *T*. We vary *T* from fifteen minutes to two hours for sensitivity analyses, where the resutls corroborate the stability of *T* (see Supplementary Fig. 7 for more details). In the article, we present the results when *T* equals to two hours for an illustration.

### The construction of the temporal contact graph

An individual has four status: susceptible, contacted, infectious and confirmed. The status ‘susceptible’ turns to ‘contacted’ when an individual had at least one contact with infectious individuals. A contacted individual may be infected or stay healthy. The status ‘infectious’ changes to ‘confirmed’ when confirmation is made. In China, confirmed cases will be quarantined for treatment and no longer infectious to others.

Recent results indicated that an infected individual can turn to infectious before and after the symptom onset, known as pre-symptomatic transmission and symptomatic transmission. Taking into account both types of transmission, we define the infectious period from the time when an infected individual becomes infectious to the time when he/she is removed (recovered or quarantined for treatment). We analyze the range of this period, finding that 17 days is the best choice (see Supplementary Fig. 6 for sensitivity analyses).

By using the contact model, we identify 519,400 susceptible individuals having contacts with 14,198 infectious individuals who turn to confirmed status later. The daily temporal contact graph is constructed as a temporal undirected weighted bipartite graph where the vertices represent contacted susceptible individuals or infectious individuals and the weight represents the number of contacts between them in a single day. This bipartite temporal graph is used in all the analysis in this article.

### Bayesian framework

We calculate the posterior probability *P*(*Z*∣*B*, *F*) under the Bayesian framework, where we denote the behavior events by *B* and denote the feature events by *F*. Specifically, *b*_*j*_ indicates the numbers of contact events for any contacted individual *j*, and *f*_*j*_ indicates the category of feature event for *j*. To measure the infection risk of a contacted individual *j*, we employ the Bayesian formula

2 $$P({z}_{j}=1| {b}_{j},{f}_{j})=\frac{P({b}_{j},{f}_{j}| {z}_{j}=1)\cdot P({z}_{j}=1)}{P({b}_{j},{f}_{j})}.$$

The term *P*(*b*_*j*_, *f*_*j*_∣*z*_*j*_ = 1) is called the likelihood, indicating the distributions of behaviors and features for any infected individual *j*. Assuming the behaviors and features are independent^53^, we have

3 $$P({b}_{j},{f}_{j}| {z}_{j}=1)=P({b}_{j}| {z}_{j}=1)\cdot P({f}_{j}| {z}_{j}=1).$$

Since we have found that the probabilities for various contacts can be approximated by power-law distributions, i.e.,

4 $$P({b}_{j}=k| {z}_{j}=1)=c\cdot {k}^{-\gamma },\,k=1,2,\cdots \,,$$

where coefficient *c* is the normalizing constant, satisfying

5 $$c=\frac{1}{\int\nolimits_{k = 1}^{\infty }{k}^{-\gamma }dk}=\gamma -1,\gamma\; > \; 1.$$

We next try to compute the values of *c* and *γ* by maximum likelihood estimate^54^. Supposing we have *N* infected samples *b*_1_, *b*_2_, ⋯  , *b*_*N*_, we obtain the likelihood function

6 $$l(\gamma )=\ln P({b}_{1},{b}_{2},\cdots \,,{b}_{N}| \gamma )=\ln \mathop{\prod }\limits_{j=1}^{N}(\gamma -1)\cdot {b}_{j}^{-\gamma }=(-\gamma )\cdot \mathop{\sum }\limits_{j=1}^{N}\ln {b}_{j}+N\cdot \ln (\gamma -1).$$

Then,

7 $$\frac{\partial l(\gamma )}{\partial \gamma }=-\mathop{\sum }\limits_{j=1}^{N}\ln {b}_{j}+N\cdot \frac{1}{\gamma -1}.$$

Holding $\frac{\partial l(\gamma )}{\partial \gamma }=0$, we can obtain

8 $$\hat{\gamma }=1+\frac{N}{\mathop{\sum }\nolimits_{j = 1}^{N}\ln {b}_{j}}.$$

As *P*(*f*_*j*_∣*z*_*j*_ = 1) indicates the features for any infected individual *j* such as gender or age, we assume the distributions are multinomial, i.e.,

9 $$P({f}_{j}=k| {z}_{j}=1)=Q(k).$$

Specifically, supposing we have *M* infected samples *f*_1_, *f*_2_, ⋯  , *f*_*M*_, the multinomial distribution *Q*(*k*) is estimated by

10 $$\hat{Q(k)}=\frac{{{{{{{{{\bf{1}}}}}}}}}_{\{{f}_{j} = k\}}}{M}.$$

Notice that there is difference between the behaviors of the infected contacted individuals and the uninfected contacted individuals. We thus denote the estimations from the infected samples by ${\hat{\gamma }}_{I}$ for contact events, and ${\hat{Q}}_{I}$ for feature events, while we denote the estimations from the uninfected samples by ${\hat{\gamma }}_{U}$ for contact events, and ${\hat{Q}}_{U}$ for feature events. Substituting Eq. (8) and Eq. (10) into Eq. (2), we can calculate the posterior probability

11 $$P({z}_{j}=1| {b}_{j},{f}_{j})=\frac{({\hat{\gamma }}_{I}-1)\cdot {b}_{j}^{-{\hat{\gamma }}_{I}}\cdot {\hat{Q}}_{I}({f}_{j})\cdot \rho }{({\hat{\gamma }}_{I}-1)\cdot {b}_{j}^{-{\hat{\gamma }}_{I}}\cdot {\hat{Q}}_{I}({f}_{j})\cdot \rho +({\hat{\gamma }}_{U}-1)\cdot {b}_{j}^{-{\hat{\gamma }}_{U}}\cdot {\hat{Q}}_{U}({f}_{j})\cdot (1-\rho )},$$

where *ρ* can be obtained by the proportion of the infectious among the population.

### Risk evaluation

In this article we have considered the risk by their behaviors and features, and we use true/false positive and the ROC curve to analyze the effectiveness of the risk model. Notice that the "positive” in the phrase "true/false positive rates” does not indicate the "positive” in a nucleic acid testing. In fact, we have measured the risk of any contacted individual *j*, i.e., *P*(*z*_*j*_ = 1) by the proposed risk model. In order to evaluate the risk measured by the model, we study the ROC (receiver operating characteristic) curve. For a threshold 0 < *q* < 1, specifically, a contacted individual *j* is considered to be true positive if *z*_*j*_ = 1 and *P*(*z*_*j*_ = 1) > *q*, while *j* is considered to be false positive if *z*_*j*_ = 0 and *P*(*z*_*j*_ = 1) > *q*. Then, we can calculate the TPR (true positive rate) by

12 $${{{{{\rm{TPR}}}}}}=\frac{{\sum }_{j}{{{{{{{{\bf{1}}}}}}}}}_{\{{{{{{{{{\bf{z}}}}}}}}}_{{{{{{{{\bf{j}}}}}}}}} = {{{{{{{\bf{1}}}}}}}},{{{{{{{\bf{P}}}}}}}}({{{{{{{{\bf{z}}}}}}}}}_{{{{{{{{\bf{j}}}}}}}}} = {{{{{{{\bf{1}}}}}}}}) \,{ > }\,{{{{{{{\bf{q}}}}}}}}\}}}{{\sum }_{j}1},$$

and FPR (false positive rate) by

13 $${{{{{\rm{FPR}}}}}}=\frac{{\sum }_{j}{{{{{{{{\bf{1}}}}}}}}}_{\{{{{{{{{{\bf{z}}}}}}}}}_{{{{{{{{\bf{j}}}}}}}}} = {{{{{{{\bf{0}}}}}}}},{{{{{{{\bf{P}}}}}}}}({{{{{{{{\bf{z}}}}}}}}}_{{{{{{{{\bf{j}}}}}}}}} = {{{{{{{\bf{1}}}}}}}}) \,{ > }\,{{{{{{{\bf{q}}}}}}}}\}}}{{\sum }_{j}1}.$$

Thus, the ROC curve described by TPR and FPR can well evaluate the risk model for contacted individuals.

### Ethics statement

The original data of location-related information was uploaded to location-based services provider, when a smartphone user requests location-based services. Smartphone users were informed the data collection process, and authorized the process once using location-based services. Westlake Institute for Data Intelligence provided the anonymous temporal contact graph. The whole process of constructing the temporal contact graph was conducted and calculated by third-party secure data servers of the local institutions. The anonymous temporal contact graph was output for research, and there is no private identifying information about the individuals accessible to us. The whole project was reviewed and approved by the Medical Ethics Committee of School of Medicine, Zhejiang University. The ethical committee did not deem it necessary to request an additional informed consent by the participants, since there is no private identifying information about the individuals accessible to us (researchers) and no interaction between the individuals and us. Moreover, the project utilized anonymous data, and did not involve evaluation of experimental or patient data. Westlake Institute for Data Intelligence aggregated mobility travel flows were previously anonymized in compliance with Civil Code of the People’s Republic of China and General Data Protection Regulation (GDPR) enforced by the European Union (see Supplementary Note III for more details about ethic and privacy issues).

### Reporting summary

Further information on research design is available in the Nature Research Reporting Summary linked to this article.

## Supplementary information

Supplementary Information

Reporting Summary

## Data Availability

The anonymous constructed temporal contact graph is available at https://github.com/MinchengWu/temporal-contact-graph, and the daily symptomatic cases are referred to the ref. ^40^.

## Code availability

Code reproducing the key results of this paper is available from the corresponding author upon reasonable request.

## References

[1] World Health Organization. Coronavirus disease (COVID-19) outbreak situation. https://www.who.int/emergencies/diseases/novel-coronavirus-2019 (2020).

[2] Kissler SM, Tedijanto C, Goldstein E, Grad YH, Lipsitch M (2020). Projecting the transmission dynamics of sars-cov-2 through the postpandemic period. Science.

[3] Australia Government Department of Health. COVIDSafe app. https://www.health.gov.au/resources/apps-and-tools/covidsafe-app (2020).

[4] Singapore Government. Tracetogether, safer together. https://www.tracetogether.gov.sg/ (2020).

[5] Nature Editorial. Show evidence that apps for COVID-19 contact-tracing are secure and effective. https://www.nature.com/articles/d41586-020-01264-1 (2020). 10.1038/d41586-020-01264-1 32350479

[6] Ferretti, L. et al. Quantifying sars-cov-2 transmission suggests epidemic control with digital contact tracing. *Science* **368**, eabb6936 (2020). 10.1126/science.abb6936 PMC7164555 32234805

[7] Firth, J. A. et al. Using a real-world network to model localized COVID-19 control strategies. *Nat. Med.* **26**, 1616–1622 (2020). 10.1038/s41591-020-1036-8 32770169

[8] Vaughan, A. There are many reasons why COVID-19 contact-tracing apps may not work. https://www.newscientist.com/article/2241041/ (2020).

[9] Hinch, R. et al. Effective configurations of a digital contact tracing app: a report to nhsx. *Technical Report* (2020).

[10] Ting DSW, Carin L, Dzau V, Wong TY (2020). Digital technology and covid-19. Nat. Med..

[11] Ballouz, T. et al. Individual-Level Evaluation of the Exposure Notification Cascade in the SwissCovid Digital Proximity Tracing App: Observational Study. *JMIR Public Health and Surveillance* **8**, e35653 (2022). 10.2196/35653 PMC9122110 35476726

[12] Rodríguez P (2021). A population-based controlled experiment assessing the epidemiological impact of digital contact tracing. Nat. Commun..

[13] Fancourt, D. et al. Covid-19 social study. *Results release* **22** (2020).

[14] Menges, D. et al. A data-driven simulation of the exposure notification cascade for digital contact tracing of SARS-CoV-2 in Zurich, Switzerland. *JAMA network open* **4**, e218184 (2021). 10.1001/jamanetworkopen.2021.8184 PMC8087953 33929521

[15] Burdinski, A., Brockmann, D. & Maier, B. F. Digital contact tracing contributes little to covid-19 outbreak containment. Preprint at *MedRxiv*10.1101/2021.06.21.21259258 (2021).

[16] Lewis, D. Why many countries failed at covid contact-tracing-but some got it right. *Nature* **588**, 384–387 (2020). 10.1038/d41586-020-03518-4 33318682

[17] Cebrian M (2021). The past, present and future of digital contact tracing. Nat. Electron..

[18] Akinbi A, Forshaw M, Blinkhorn V (2021). Contact tracing apps for the covid-19 pandemic: a systematic literature review of challenges and future directions for neo-liberal societies. Health Inf. Sci. Syst..

[19] Elmokashfi, A. et al. Nationwide rollout reveals efficacy of epidemic control through digital contact tracing. *Nat. Commun*. **12**, 5918 (2021). 10.1038/s41467-021-26144-8 PMC8505561 34635661

[20] He, S. et al. Near-optimal allocation algorithms for location-dependent tasks in crowdsensing. *IEEE Trans. Vehicular Technol*. **66**, 3392–3405 (2017).

[21] Sun, K., Chen, J. & Viboud, C. Early epidemiological analysis of the coronavirus disease 2019 outbreak based on crowdsourced data: a population-level observational study. *Lancet Digital Health* **2**, E201–E208 (2020). 10.1016/S2589-7500(20)30026-1 PMC7158945 32309796

[22] Chinazzi, M. et al. The effect of travel restrictions on the spread of the 2019 novel coronavirus (COVID-19) outbreak. *Science* **368**, 395–400 (2020). 10.1126/science.aba9757 PMC7164386 32144116

[23] Kraemer, M. U. G. et al. The effect of human mobility and control measures on the COVID-19 epidemic in China. *Science* **368**, 493–497 (2020). 10.1126/science.abb4218 PMC7146642 32213647

[24] Wu JT (2020). Estimating clinical severity of COVID-19 from the transmission dynamics in Wuhan, China. Nat. Med..

[25] Jia, J. S. et al. Population flow drives spatio-temporal distribution of COVID-19 in China. *Nature* **582**, 389–394 (2020). 10.1038/s41586-020-2284-y 32349120

[26] Tong, Z.-D. et al. Potential presymptomatic transmission of sars-cov-2, Zhejiang province, China. *Emerging Infect. Dis*. **26**, 1052–1054 (2020). 10.3201/eid2605.200198 PMC7181913 32091386

[27] Bai, Y. et al. Presumed asymptomatic carrier transmission of COVID-19. *Jama* **323**, 1406–1407 (2020). 10.1001/jama.2020.2565 PMC7042844 32083643

[28] Zhao, S. et al. Preliminary estimation of the basic reproduction number of novel coronavirus (2019-ncov) in China, from 2019 to 2020: a data-driven analysis in the early phase of the outbreak. *Int. J. Infect. Dis*. **92**, 214–217 (2020). 10.1016/j.ijid.2020.01.050 PMC7110798 32007643

[29] He, X. et al. Temporal dynamics in viral shedding and transmissibility of COVID-19. *Nat. Med*. **26**, 672–675 (2020). 10.1038/s41591-020-0869-5 32296168

[30] Chowell, G., Cleaton, J. M. & Viboud, C. Elucidating transmission patterns from internet reports: Ebola and middle east respiratory syndrome as case studies. *J. Infect. Dis*. **214**, S421–S426 (2020). 10.1093/infdis/jiw356 PMC5144900 28830110

[31] Wu, J. T., Leung, K. & Leung, G. M. Nowcasting and forecasting the potential domestic and international spread of the 2019-ncov outbreak originating in wuhan, china: a modelling study. *Lancet* **395**, 689–697 (2020). 10.1016/S0140-6736(20)30260-9 PMC7159271 32014114

[32] Lipsitch, M. et al. Transmission dynamics and control of severe acute respiratory syndrome. *Science* **300**, 1966–1970 (2003). 10.1126/science.1086616 PMC2760158 12766207

[33] Riley, S. et al. Transmission dynamics of the etiological agent of sars in hong kong: impact of public health interventions. *Science* **300**, 1961–1966 (2003). 10.1126/science.1086478 12766206

[34] Shaman, J., Karspeck, A., Yang, W., Tamerius, J. & Lipsitch, M. Real-time influenza forecasts during the 2012–2013 season. *Nat. Commun*. **4**, 2837 (2013). 10.1038/ncomms3837 PMC3873365 24302074

[35] Kucharski, A. J. et al. Early dynamics of transmission and control of COVID-19: a mathematical modelling study. *Lancet Infect. Dis*. **20**, 553–558 (2020). 10.1016/S1473-3099(20)30144-4 PMC7158569 32171059

[36] Pastor-Satorras, R. & Vespignani, A. Epidemic spreading in scale-free networks. *Phys. Rev. Lett*. **86**, 3200 (2001). 10.1103/PhysRevLett.86.3200 11290142

[37] Kitsak, M. et al. Identification of influential spreaders in complex networks. *Nat. Phys*. **6**, 888–893 (2010).

[38] Wu M (2019). A tensor-based framework for studying eigenvector multicentrality in multilayer networks. Proc. Natl. Acad. Sci. USA.

[39] Aleta, A. et al. Modeling the impact of testing, contact tracing and household quarantine on second waves of COVID-19. *Nat. Hum. Behav.* **4**, 964–971 (2020). 10.1038/s41562-020-0931-9 PMC7641501 32759985

[40] Hao X (2020). Reconstruction of the full transmission dynamics of covid-19 in wuhan. Nature.

[41] He X (2020). Temporal dynamics in viral shedding and transmissibility of covid-19. Nat. Med..

[42] Linton NM (2020). Incubation period and other epidemiological characteristics of 2019 novel coronavirus infections with right truncation: a statistical analysis of publicly available case data. J. Clin. Med..

[43] Lauer SA (2020). The incubation period of coronavirus disease 2019 (covid-19) from publicly reported confirmed cases: estimation and application. Ann. Intern. Med..

[44] Sohrabi, C. et al. World health organization declares global emergency: a review of the 2019 novel coronavirus (COVID-19). *Int. J. Surgery* **76**, 71–76 (2020). 10.1016/j.ijsu.2020.02.034 PMC7105032 32112977

[45] Li, Q. et al. Early transmission dynamics in wuhan, china, of novel coronavirus–infected pneumonia. *N. Engl. J. Med*. **382**, 1199–1207 (2020). 10.1056/NEJMoa2001316 PMC7121484 31995857

[46] Bi, Q. et al. Epidemiology and transmission of covid-19 in 391 cases and 1286 of their close contacts in shenzhen, china: a retrospective cohort study. *Lancet Infect. Dis*. **20**, 911–919 (2020). 10.1016/S1473-3099(20)30287-5 PMC7185944 32353347

[47] Cao, M. et al. Clinical features of patients infected with the 2019 novel coronavirus (COVID-19) in Shanghai, China. Preprint at *MedRxiv*10.1101/2020.03.04.20030395 (2020).

[48] Chen, J. et al. Clinical progression of patients with COVID-19 in Shanghai, China. *J. Infect*. **80**, e1–e6 (2020). 10.1016/j.jinf.2020.03.004 PMC7102530 32171869

[49] Cheng, Y. et al. Kidney disease is associated with in-hospital death of patients with COVID-19. *Kidney Int*. **97**, 829–838 (2020). 10.1016/j.kint.2020.03.005 PMC7110296 32247631

[50] Bernardo, J. M. & Smith, A. F. *Bayesian Theory*, vol. 405 (John Wiley & Sons, 2009).

[51] Giordano, G. et al. Modelling the COVID-19 epidemic and implementation of population-wide interventions in italy. *Nat. Med*. **26**, 855–860 (2020). 10.1038/s41591-020-0883-7 PMC7175834 32322102

[52] Li W (2022). A spatiotemporal decay model of human mobility when facing large-scale crises. Proc. Natl. Acad. Sci. USA.

[53] Flach PA, Lachiche N (2004). Naive bayesian classification of structured data. Mach. Learn..

[54] Bauke H (2007). Parameter estimation for power-law distributions by maximum likelihood methods. Eur. Phys. J. B.

