# Supplementary material for: Use of temporal contact graphs to understand the evolution of COVID-19 through contact tracing data
Source: Commun Phys. 2022 Nov 4;5(1):270. doi: 10.1038/s42005-022-01045-4 (PMC9638278; doi:10.1038/s42005-022-01045-4)
Supplement: Supplementary file 1 — Supplementary Information [file 42005_2022_1045_MOESM1_ESM.pdf]

Supplementary Information for

Use of temporal contact graphs to understand the evolution of COVID-19 through contact tracing data

Mincheng Wu[1,†], Chao Li[1,†], Zhangchong Shen[1], Shibo He[1,2,*], Lingling Tang[3], Jie Zheng[4], Yi Fang[5], Kehan Li[1], Yanggang Cheng[1], Zhiguo Shi[2,6], Guoping Sheng[3], Yu Liu[2,5], Jinxing Zhu[5], Xinjiang Ye[5], Jinlai Chen[2,5], Wenrong Chen[5], Lanjuan Li[7,*], Youxian Sun[1], Jiming Chen[1,2,*]

[1]*College of Control Science and Engineering, Zhejiang University, Hangzhou 310027, China.*
[2]*Data Intelligence Research Center, Institute of Wenzhou, Zhejiang University, Wenzhou, China.*
[3]*Shulan (Hangzhou) Hospital Affiliated to Shulan International Medical College, Zhejiang Shuren University, Hangzhou, China.*
[4]*Zhejiang Institute of Medical-care Information Technology, Hangzhou, China.*
[5]*Westlake Institute for Data Intelligence, Hangzhou, China.*
[6]*College of Information Science and Electronic Engineering, Zhejiang University, Hangzhou, China.*
[7]*State Key Laboratory for Diagnosis and Treatment of Infectious Diseases, Zhejiang University, Hangzhou, China.*

[*] Corresponding author. E-mail:s18he@zju.edu.cn, ljli@zju.edu.cn, cjm@zju.edu.cn.

[†] These authors contributed equally.

Supplementary Note I: Data description

The original data of location-related information was collected by location-based service (LBS) providers in China, which have a long-term cooperation agreements with Westlake Institute for Data Intelligence (the affiliate of some coauthors of this article). The location-related information was uploaded every time smartphone users are using LBS. Smartphone users authorized such data collection process. According to the Personal Information Security Specification of China (2019), privacy protection mechanisms such as perturbation and pseudonymization are adopted during data collection. Based on Public Health Emergencies Regulations of China, the local authority provided the device identifications of confirmed cases. Such information can be incorporated to link the health status of smartphone users since the app accounts in China are registered by these identifications.

Many recent works used very similar data collected from different sources. For example, Kraemer et al. evaluated how the individual mobility affects the disease transmission, and, as control measures can be reflected by the changes in individual mobility, further showed the positive effect of control measures in Wuhan on mitigating the spread of COVID-19[1]. Nicholas et al. used country-wide aggregated mobile phone data to show that the outflow from Wuhan accurately predicts the distribution of infections across all of China, and developed a "risk source" model to forecast confirmed cases and identify high risk locales at early stage. This work also derived the geographic spread and growth pattern of COVID-19[2]. Vespignani et al. used the data collected by Cuebiq Inc[3], which is similar with the location data we have. However, they simulate the infectious cases using a stochastic discrete-time compartmental model, which is not calibrated to account for the specific evolution in Boston. From the above summary, we can see that we utilized the real health status of users to construct the temporal contact graph, and provides an empirical evidence to reveal evolving epidemic situation of COVID-19. These distinguishes our work from existing ones. In other words, we took the first attempt to construct the contact graph between susceptible and infectious individuals to represent the process of digital contact tracing, while previous studies did not utilized the information of confirmed cases.

We note that all individual location-related data and health status information were collected, stored, and used by following the Personal Information Security Specification (2019) and Public Health Emergencies Regulations of China. All raw data was stored in specialized data servers with limited access by LBS providers. For the research purpose, the Westlake Institute for Data Intelligence constructed the temporal contact graph according to the contact model we built and

provided it for further analysis. All the results in the article can be obtained and validated by the temporal contact graph, which does not contain private geographic and identity information about the contacted individuals and the confirmed cases. This project was reviewed and approved by the Medical Ethics Committee of School of Medicine, Zhejiang University.

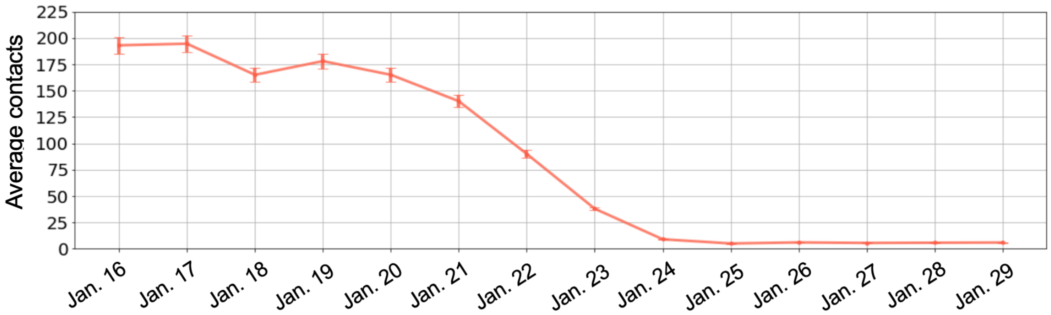

Supplementary Figure 1: **Average number of contacts of the entire population.** Before and after the travel restriction of the general population daily contact number changes.

Supplementary Fig. **1** shows the average contact trends of the whole population (10,527,737 smartphone users) in Wuhan during the period from 16 to 29 January, 2020. After the Chinese authority confirmed the COVID-19 coronavirus can be transmitted among human on 20 January, 2020, the average daily contacts of the whole population dropped sharply. After the ban of non-essential vehicles in Wuhan downtown area on 26 January, 2020, the average contacts reached the lowest, and the average contacts stayed stable at such a level for a long period.

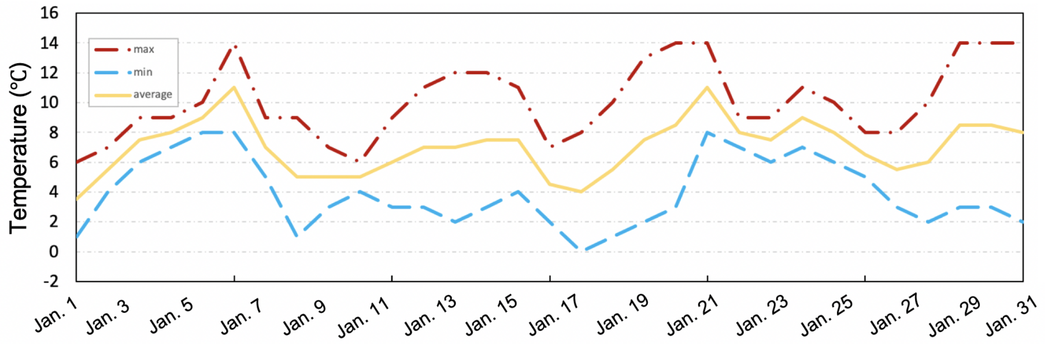

Supplementary Figure 2: **Daily temperature in Wuhan from 1 January, 2020 to 31 January, 2020.** The weather data comes from meteoblue.com.

Supplementary Note II: Statistical analysis

Constructed graph structure

In the article we have used five indicators by leveraging the temporal contact graph: the total number of daily contacts $C(t)$ by calculating the number of edges of the temporal contact graph in day t, the total number of active infectious individuals $I(t)$, the number of susceptible individuals $S(t)$ who had at least one contact with infectious individuals, the average number of susceptible individuals $k_I(t)$ that each infectious individual contacted in day t (by calculating the average degree of the nodes representing infectious individuals in the graph), the number of infectious individuals $k_S(t)$ that each susceptible individual encountered in day t (by calculating the average degree of nodes representing the susceptible). A toy example is shown for specific calculation (Supplementary Fig. **3**).

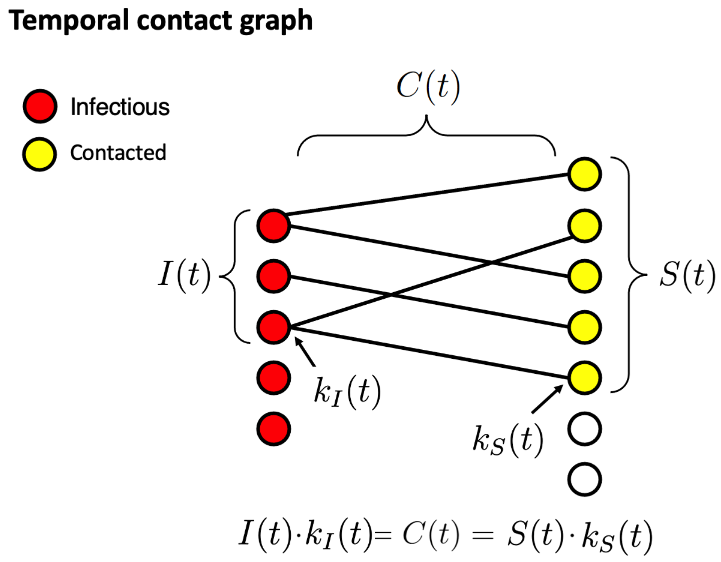

Supplementary Figure 3: **A toy example for a snapshot (one day) of the temporal contact graph.** Nodes on the left are infectious individuals and the nodes on the right are susceptible individuals. An edge indicates a contact between the two individuals in this day. The total contacts $C(t)$ is the number of edges in the bipartite graph, i.e., $C(t) = 5$. The number of active infectious individuals equals three, i.e., $I(t) = 3$, while the number of contacted individuals equals five, i.e., $S(t) = 5$. Also, we can calculate that $k_I(t) = 5/3$ and $k_S(t) = 1$.

Since the fraction of the infectious individuals in a population is very small, $k_I(t)$ is much

larger than $k_S(t)$ generally. For example, in the contact graph $G(t)$, $k_S(t) = 1$ while $k_I(t) = 5$ (Supplementary Figure **4**). As a matter of fact, the two metrics are on very different orders of magnitude, since the contact graph is much larger than the toy example. The units for $k_S(t)$ and $k_I(t)$ are people/day. Thus, once the temporal contact graph is constructed by the contact model, the five informative indicators can be determined.

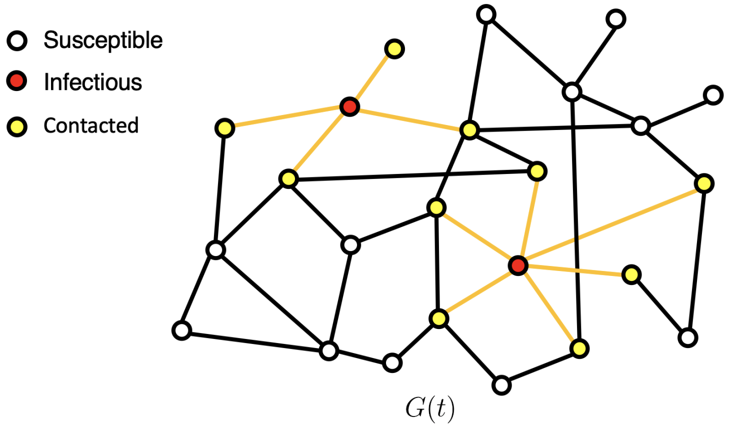

$G(t)$

Supplementary Figure 4: **A toy example for a snapshot of the spreading.** The spreading in day t is represented by the graph $G(t)$, where nodes are individuals and edges are contacts between two individuals. We denote different health statuses by distinct colors, and the contacts between infectious and contacted individuals are colored by orange.

Infectious period

As mentioned in the Supplementary Note I, the collected data include location-based information. Based on the collected data, we use a contact model, quantifying a contact between two individuals when they are geographically close to each other. Specifically, since locations of individuals is perturbed, we define a contact occurring when two individuals are in the same perturbed area within a given time interval. Based on the proposed contact model, we constructed the temporal contact graph, which describes the daily contacts between the susceptible and infectious individuals: a node in the graph means a susceptible or infectious individual, and an edge in day t indicates a contact occurring in day t between a susceptible individual and infectious individual.

However, different model parameters do impact the measurements for the contact such as the infectious period and time interval in the contact model. Since the infectious period is an important parameter to determine the temporal contact graph, we perform sensitivity analyses for this period from 10 days to 30 days (Supplementary Figure **5**). Although the absolute values of the

five indicators change, we can see that the trends remain stable.

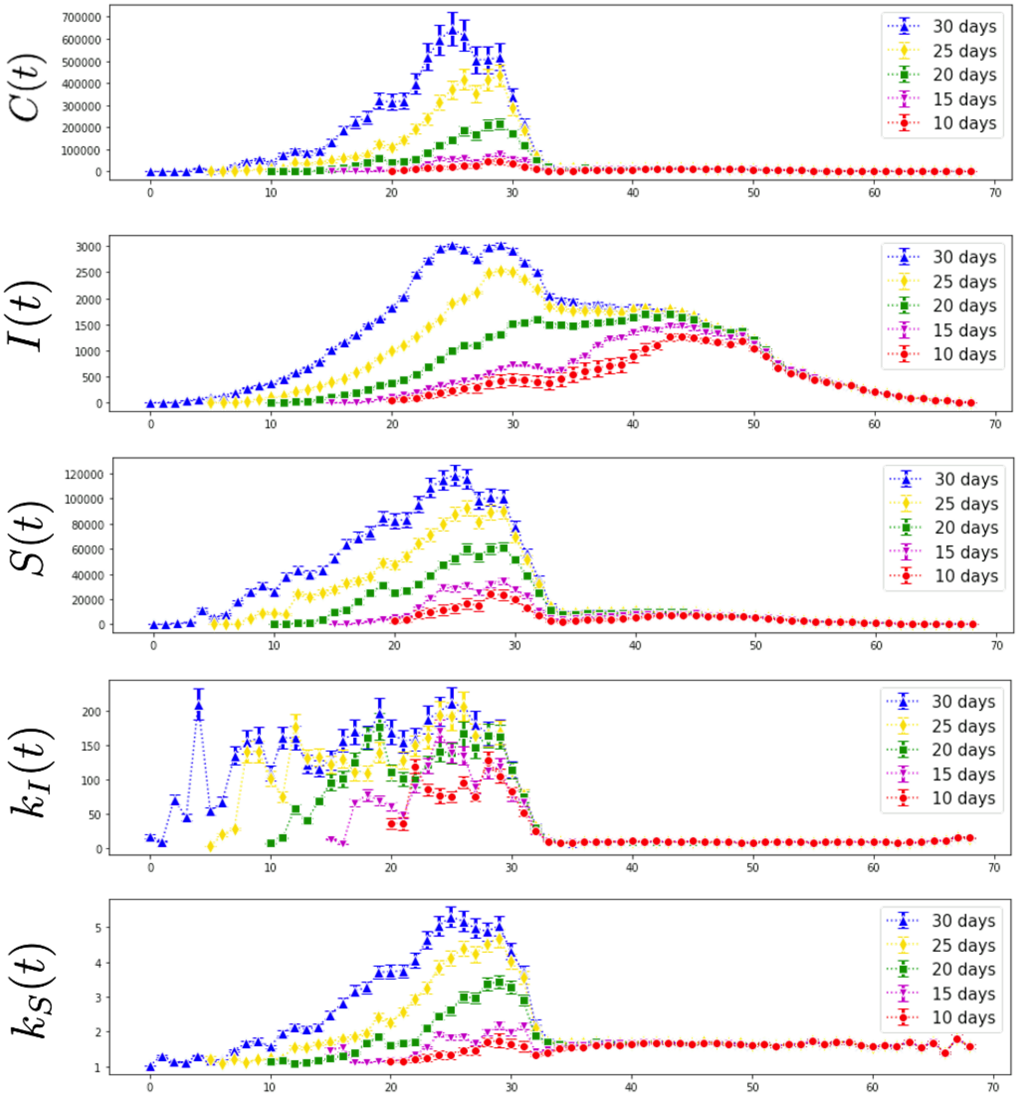

Supplementary Figure 5: **Sensitivity analysis for the infectious period a.** $C(t)$, the daily total numbers of contacts between infectious individuals and contacted individuals. **b.** $I(t)$, the daily total number of infectious individuals that have contacts with contacted individuals at least once. **c.** $S(t)$, the daily total number of contacted individuals that have contacts with infectious individuals at least once. **d.** $k_I(t)$, the daily average numbers of contacts with contacted individuals for infectious individuals before confirmation. **e.** $k_S(t)$, the daily average numbers of contacts with infectious individuals for contacted individuals.

Further, we calculate the Pearson correlations between daily contacts and infectious individ-

uals with delays ranging from 5 to 30 days for different infectious period (Supplementary Figure **6**). Obviously, the experiments show that a 23-day delay (between the number of daily confirmed cases and daily total contacts) results in the best Pearson correlation of 0.73, where the corresponding infectious period equals 17 days in accordance with existing surveys[4–11]. Thus, we consider the infectious period to be 17 days in the article, and an individual is identified as infectious in day t if he/she was confirmed during $[t+1, t+17]$.

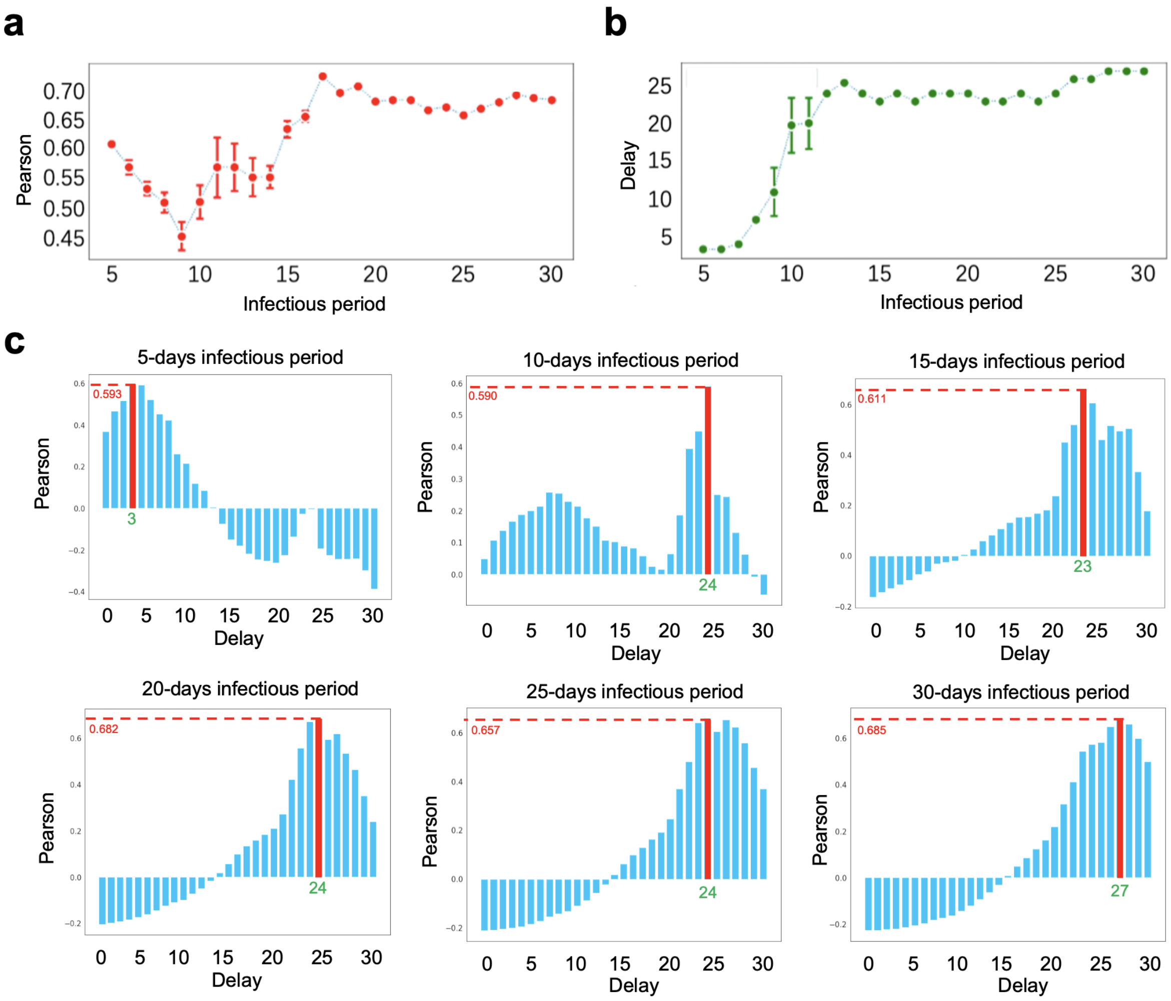

Supplementary Figure 6: **Sensitivity for the infectious period.** We calculate the Pearson correlations between the daily contacts the daily confirmed cases with a delay ranging from 0 to 30 days.

We also performed the sensitivity analysis for the contact model by varying the time interval from 15 minutes to 120 minutes and the infectious period from 1 days to 30 days in the contact

model. Specifically, we vary the time interval from 15 minutes to 120 minutes and test the contact models under different time granularities, finding that such a time granularity does not change our conclusion in this article. We find that the Pearson coefficient between the daily number of contacts and delayed daily number of confirmed cases reaches maximum when the delay is 23 days, corresponding to a 17-days infectious period for all values of time interval (Supplementary Figure **7**). This indicates that the proposed contact model is very stable.

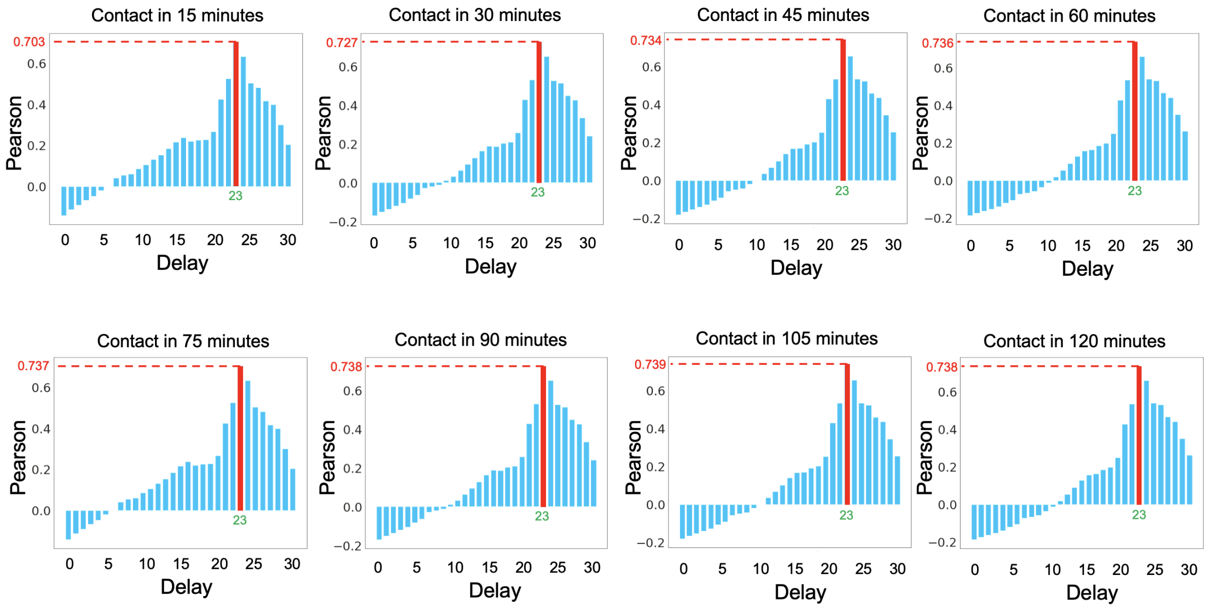

Supplementary Figure 7: **Sensitivity for the time interval** T**.** The Pearson correlations between daily contacts and daily confirmed cases with a delay ranging from 5 days to 30 days. Each panel corresponds to the time interval T for a contact in the contact model.

User involvement

To perform sensitivity analysis for the impacts of users' involvement, we further conduct experiments to simulate different user involvements by randomly selecting $\alpha\%$ users as the voluntary users, and $\alpha\%$ data items each user uploading per day, and evaluate the corresponding performance loss. To show the robustness of our analysis, we repeat 10 times Monte Carlo experiments for a given setting. Specifically, we set user involvement rate to 10%, 30%, 50%, 70%, 90%, and user uploading rate to 10%, 30%, 50%, 70%, 90%. Then, we perform the corresponding experiments. We plot the curves of $k_S(t)$, $k_I(t)$ and $C(t)$ calculated from the contact model with different uploading rates from 1 January to 28 February with error bars (Supplementary Figure **8**a). The results show that with the decrease of uploading rates, the values of three parameters decrease obviously,

which is because the number of recorded contacts drops along with decreasing upload rates. Notice that the error bars of three parameters are all small, which implies the Monte Carlo experiments are stable to produce similar results. It is, therefore, reasonable to emphasize that the number of user uploads reduced for each Monte Carlo experiment is the same, and the total number of participation does not reduce under user upload analysis. Generally speaking, the Pearson correlation between case of 10% uploading rate and other cases ($\alpha\%$ upload rates) remains high for the analysis of user uploading rate. This phenomenon implies that we might not need a high uploading rate if we are only interested in estimating the trends.

We conduct experiments in the same way as those for uploading rates to evaluate the impact of diverse participation rates (Supplementary Figure **8**b). The corresponding results show that, compared with uploading rate analysis, decreasing the participation rates brings more uncertainty in the Monte Carlo experiments with higher error bars. This is because the participants that are randomly deleted at each time experiment are different. Thus, their impacts on the entire network also vary. Notice that when the participation rate is not very small, the high correlation can still be preserved. However, when it is reduced to 10%, the correlation coefficient decreases significantly. This could be attributed to the power law distribution of the network: since the distribution has an obvious long-tail effect, only when the participation rate is low enough can some key nodes be deleted, thereby affecting the trend of the proposed metrics.

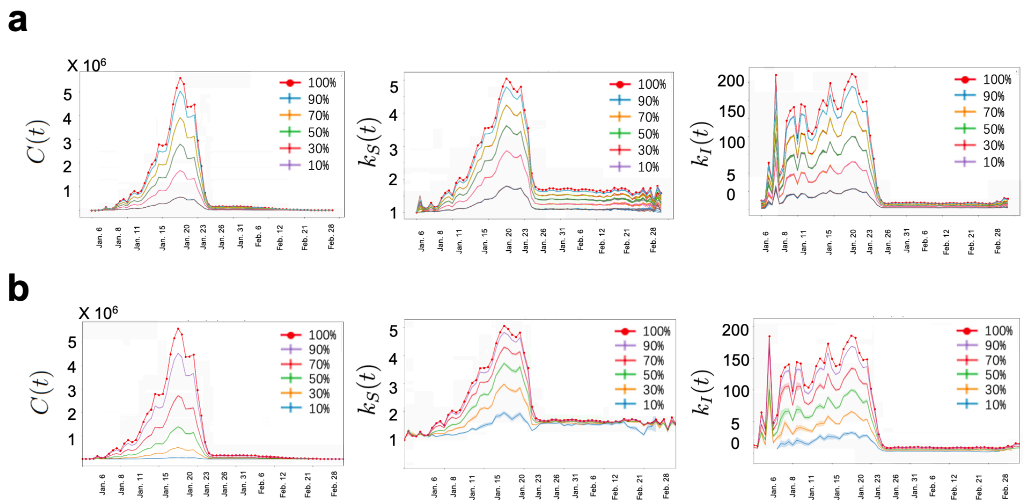

Supplementary Figure 8: **Sensitivity analysis for user involvement. a.** The proposed metrics including $C(t)$, $k_S(t)$ and $k_I(t)$ are shown, when simulating the uploading rate ranging from 10% to 90%. **b.** The proposed metrics including $C(t)$, $k_S(t)$ and $k_I(t)$ are shown, when simulating the participation rate ranging from 10% to 90%.

We have analyzed the contact model in main body of the paper with six curves showing different user participation rates and upload rates. Here we give the corresponding statistical information such as median and variances of $k_S(t)$,$k_I(t)$ and total contacts $C(t)$ shown in Supplementary Figure **9**. It reveals that as $\alpha\%$ of participation and upload rate decreases, the statistical information decreases with the similar trend. This is expected as reduction in either user participation rate or user upload rate decreases the chances of having contacts among users.

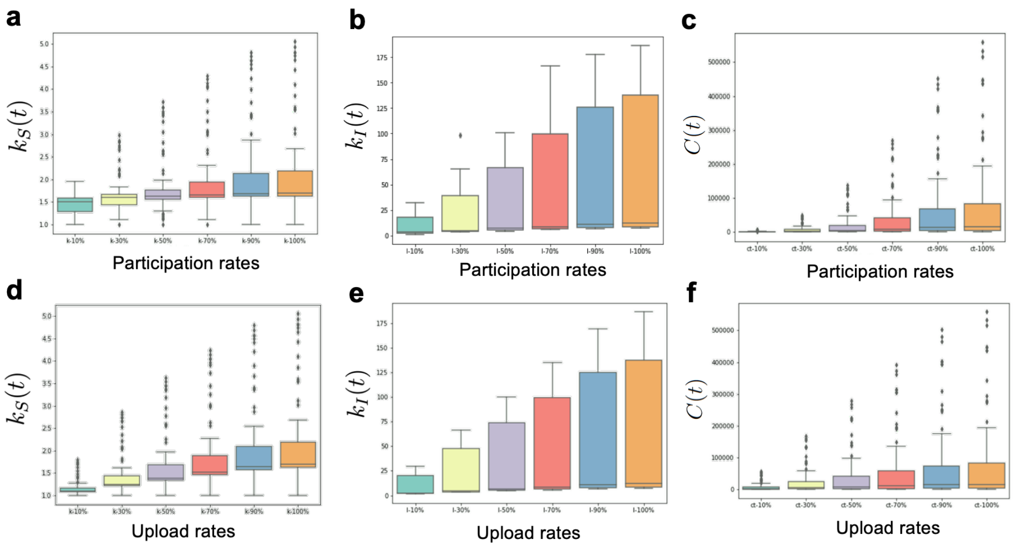

Supplementary Figure 9: **Performance of different user involvement in the contact model. a-c.** Three box plots show the distribution change of $k_S(t)$, $k_I(t)$, and daily number of total contacts Vs. different user participation rates. **d-f.** Three box plots show the distribution change of $k_S(t)$, $k_I(t)$, and daily number of total contacts Vs. different user upload rates.

Moreover, the practical challenges for real contact tracing apps are: what to do if we have only partial information about contacts and about the real individual health status? As a matter of fact, we have investigated this in this article, indicating the impacts of user involvement on the contact tracing app performance. We have conducted experiments to simulate different user involvements by randomly selecting $\alpha\%$ users as the voluntary users, and evaluate the corresponding performance loss, where $\alpha\% = 10\%, 30\%, 50\%, 70\%, 90\%$. In such a way, both susceptible and infectious individuals could be removed, which simulates partial information case about both susceptible and infectious individuals. One interesting result in our work is even though we only have partial information about the contacts and the conformed cases, we can still have a good performance on estimating the evolving situation of COVID-19 when $\alpha\%$ is not low enough.

Supplementary Note III: Data and real contagion

Data biases

In this article, we analyze the evolving epidemic situation of COVID-19, leveraging the available reported 'confirmed cases' as a proxy of the measure of the extent of the contagion However, many other factors (other than actual COVID19 transmission) may have influenced the recorded number of confirmed cases because of medical resource lacking, incomplete information and so on. We will consider how could these biases in the number of confirmed cases (compared to the number of actual cases) shape the actual results. In fact, the reported number of confirmed cases is typically less than the number of actual cases. Therefore, there is always biases in reality. However, it is impossible to know the actual cases even their distributions. Here, we assume that the reported confirmed cases $Sc(t)$ is proportional to the number of actual cases in reality $Sr(t)$, i.e.,

$$Sc(t) = \beta \cdot Sr(t), \tag{1}$$

where $0 < \beta < 1$ is a constant to quantify the probability for an actual case being confirmed. Then, in this article we perform correlation analysis between the number of daily contacts $C(t)$ and daily confirmed cases $Sc(t)$, and obtain a Pearson coefficient $\rho(C(t), Sc(t)) = 0.78$. Then, we will prove the Pearson coefficient between the daily contacts $C(t)$ and daily infected individuals in reality $Sr(t)$ is the same, i.e.,

$$\rho(C(t), Sr(t)) = \rho(C(t), \beta \cdot Sc(t)) = \rho(C(t), Sc(t)) = 0.78. \tag{2}$$

In other words, although $Sc(t)$ has a bias from $Sr(t)$, i.e., $Sc(t) = \beta \cdot Sr(t)$, it does not affect the correlation analysis in our results.

Recorded contacts and transmission

A primary conceptual focus of the manuscript is how real-world social contacts may relate to COVID-19 contagion patterns. For instance, we define a contact as a co-occurrence within a specific distance (e.g., $15m \times 29m$) in the proposed contact model, when in fact it is unlikely that this virus would be transmitted at a range even 10 fold less than this. Therefore, it is necessary to clarify how this might influence the patterns found, or the conclusions drawn. Medically speaking, a close contact is said to occur when two individuals are within a distance of 1.8 meters [13]. Individuals who have close contacts with infectious cases generally have a high probability of getting infected.

However, in practice, it is difficult to decide a close contact in a digital way. Most contact tracing apps exploit Bluetooth and/or GPS to decide a contact when two individuals are in a short distance (e.g., within 20 meters). In fact, one of the current controversial issues lies in whether such type of contacts captured by contact tracing apps is effective since it is not fine-grained enough. For example, the mentioned potential interesting work discussed the importance of network structure and social dynamics in evaluating the potential impact of SARS-CoV-2 control by combing fine-scale data[14]. However, our results show that there is still a clear distinction of contact behaviors between the infected and uninfected contacted individuals under our contact model. Therefore, the frequency of social contacts captured by contact tracing apps actually has a high correlation with contagion patterns.

Tracing apps

In this article, we use the location-related data from tracing apps to calculate if two individuals are close. Based on this, we build the temporal contact graph, which shows the contact relationship between susceptible and infectious individuals. Although the data used in our article is different with the Bluetooth based contact tracing apps, the principles used to define the potential contact are the same. By studying co-location data for 10 millions mobile phone users in Wuhan, our aim is to understand and clarify the potential of contact tracing apps to identify and interrupt transmission chains of the SARS-CoV-2 virus. However, controversies on the contact tracing apps mainly include: 1) the contact tracing apps may have privacy leakage issues and the methods (centralized vs. decentralized) used to inform the potential contacts with infectious cases about their risks need further investigation; 2) current contact tracing apps utilize location-related information (e.g., Bluetooth, WiFi or GPS) to define a contact and are not fine-grained enough to capture a close contact within a distance of 1.5 meters; 3) such apps may not work for suppressing the transmission of COVID-19 when the participation rate is not high enough. For the first concern, Google recently showed that the privacy can be protected by using Bluetooth with a proper privacy-preserving protocol. Our work here studies the contact behavior analysis in the transmission, and thereby does not discuss the privacy issues. Note that our approach falls within the category of the centralized way of informing the potential contacts. Though both centralized and decentralized ways can identify individuals having contacts with infectious cases, our results demonstrate that centralized way can provide an abundance of information that can be helpful for prevention and control of COVID-19 (see explanations for the second and third issues as follows). For the second issue, it is true that contact tracing apps cannot accurately define a close contact and may ignore other important factors that impact the spread of COVID-19, e.g., whether a protection

measure such as wearing a mask is taken in a contact. However, our results show that there is still a prominent distinction of contact behaviors between the infected and uninfected contacted individuals. Based on this, we designed an infection risk evaluation framework to identify potential infected ones. For the third issue, we evaluated the effect of user involvement and show that user participation rate exerts higher influence on situation evaluation than user upload rate does. Moreover, our results indicate that the contact tracing apps can still be helpful even when user involvement is low. Also, we find that five indicators calculated from the constructed temporal contact graph are informative to understand the actual transmission and evaluate the epidemic situation. In summary, though this article cannot solve all the controversial issues, we provide new evidence that contact tracing apps can be very helpful to the prevention and control of COVID-19.

Currently, many other papers in recent times (the company Cuebiq for example provided a lot of similar data to many researchers in Europe and USA), used data collected from GPS, POI as well as WiFi. In fact, most contact tracing apps exploit Bluetooth and/or GPS on smartphones to discover nearby devices held by users and identify the contacts between the susceptible and infectious individuals. For example, most contact tracing apps defined a contact by a Bluetooth "handshake", which occurs for two individuals within a distance of 20 meters. Some other apps used additional data such as GPS for contact tracing, e.g., Norway released an app that collects both GPS and Bluetooth information. In this article, a contact is identified for two individuals within about 20 meters. Clearly, our contact model works in a very similar way to those defined by most of the contact tracing apps, though the data is not directly collected from contact tracing apps (which is not available). To summarize, although the data used in our paper is different with the Bluetooth contact tracing apps, the principles used to define the potential contact are just the same.

Ethic and privacy issues

Since many ethic issues have been raised, it is of significance to discuss the guidelines for COVID-19 tracing apps[15]. Local institutions for disease prevention and control have the authority to collect the information on COVID-19 (including the patient information) and they also have the obligation to protect the privacy of patients when processing these data according to the Law on the Prevention and Control of Infectious Diseases of the People's Republic of China. Specifically, Article 12 of the Law on the Prevention and Control of Infectious Diseases of the People's Republic of China stipulates that "All units and individuals within the territory of the People's Republic of China shall accept the preventive and control measures taken by disease prevention and control

institutions and medical agencies for investigation, testing, collection of samples of infectious diseases and for isolated treatment of such diseases, and they shall provide truthful information about the diseases. Disease prevention and control institutions and medical agencies shall not divulge any information or materials relating to personal privacy". Therefore, since the outbreak of COVID-19, local institutions have collected a large set of data. To protect the privacy of patients, these data are stored on the secure data servers owned by local institutions.

In our project, we identified the confirmed cases based on the phone number information. However, we hereby clarify that we (including authors from Westlake Institute for Data Intelligence) did not obtain any information regarding the exact phone numbers of patients. As mentioned in the first paragraph, these data are stored on the secure data servers and are not available for us. To facilitate our research, Westlake Institute for Data Intelligence uploaded the mobility data contributed by smartphone users and their account information in Wuhan from Jan. 1 to Feb. 28 to the secure data servers owned by local institutions. It is noteworthy that the phone number information here refers to the encrypted (i.e., hashed) information from the original phone number. To protect users from privacy leakage and malicious attack, their phone numbers were mapped to hash values. This procedure was irreversible and would be completed once the account was created. This is actually a popular approach adopted by many location-based service providers. The phone numbers of patients were also mapped to hash values by the same hash function on the secure data servers by local institutions. Since a phone number yielded a unique hash value, we could leverage such a connection to identify the patients. The temporal contact graph encoding the contact among the infectious and the susceptible individuals was then constructed.

A schematic diagram of the above procedure is shown in Figure **10**. The whole process of constructing the temporal contact graph was conducted on the secure data servers of the local institutions. In the temporal contact graph, each node (a user) is further anonymized and thus cannot be traced back to his/her phone number information (i.e., hashed values) anymore. This means that we were unable to gain access to any identifiable phone number or hash value about each node in the temporal contact graph even though some nodes were identified as confirmed cases. Moreover, the mobility data uploaded by Westlake Institute for Data Intelligence were destroyed on the secure data server, and the temporal contact graph was provided for us offline only for research purpose. Since Westlake Institute for Data Intelligence does not own the phone numbers of confirmed cases and the temporal contact graph does not contain any private information, we do not have the authority issue. We agree that the phone number is a unique identifier. However, it was well protected in our research, since the phone numbers of confirmed cases would be hashed before

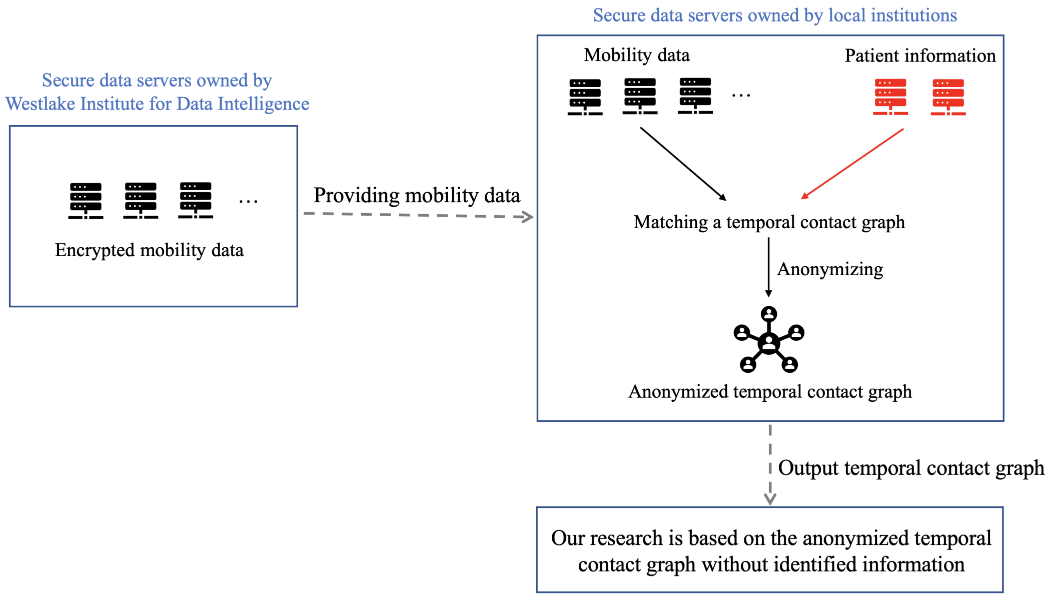

Supplementary Figure 10: **The procedure of constructing a temporal contact graph.**

they were used on the secure data servers. Furthermore, such information was securely stored on the data servers of local institutions and it was not available for us. We merely utilized the temporal contact graph to obtain relevant results in our current research. This is what we meant by saying "did not include identified individual-level data". As mentioned above, both the personal phone number data from Westlake Institute for Data Intelligence and those from the local institutions were preprocessed by the same pseudonymization mechanism (i.e., hash function). Besides, the final output (i.e., the temporal contact graph) was further pseudonymized to avoid data restoration. Thus, the hashed phone numbers do not allow individuals to be identified as covid-19 patients or individually identified. In a word, there is no private identifying information about the individuals accessible to us (researchers) and no interaction (or intervention) between the individuals and us. In addition, we note that the whole procedure is amenable to laws in both China and EU as follows. Article 1038 of the Civil Code of the People's Republic of China, stipulates that "Information processors shall not disclose or tamper with the personal information they collect or store; and without the consent of the data subject, information processors shall not illegally provide any other person with his personal information, except for the processed information cannot be identified with any specific person and restored." Article 89 of the General Data Protection Regulation (GDPR) enforced by the European Union stipulates that "Processing for archiving purposes in the public interest, scientific or historical research purposes or statistical purposes, shall be subject to appropriate safeguards, in accordance with this Regulation, for the rights and freedoms of the data subject. Those safeguards shall ensure that technical and organizational measures are in place in

particular in order to ensure respect for the principle of data minimization. Those measures may include pseudonymization provided that those purposes can be fulfilled in that manner. Where those purposes can be fulfilled by further processing which does not permit or no longer permits the identification of data subjects, those purposes shall be fulfilled in that manner."

Another key point in the debate on the contact tracing apps is about the concerns that the contact tracing apps may have privacy leakage issues and the methods (centralized vs. decentralized) used to inform the potential contacts with infectious cases about their risk need further investigation. For this issue, Google recently showed that the privacy can be protected by using Bluetooth with a proper privacy-preserving protocol[16]. Also, there are some other works on this direction[17–19]. Our work here studies the contact analysis in the transmission, and thereby does not discuss the privacy issues. Note that our approach falls within the category of the centralized way of informing the potential contacts. Though both centralized and decentralized ways can identify individuals having contacts with infectious cases, our results demonstrate that centralized way can provide an abundance of information that can be helpful for prevention and control of COVID-19. As indicated by the title of our article, our main focus is to reveal the evolving situation of COVID-19 by exploiting the temporal contact graph. The study of the effects that an app will have on people's behaviour is another important topic. There are also some works on this direction[20,21]. This topic is a new and independent topic and therefore was not considered in this work.

Supplementary References

1. Kraemer, M. U. *et al.* The effect of human mobility and control measures on the covid-19 epidemic in china. *Science* **368**, 493–497 (2020).

2. Jia, J. S. *et al.* Population flow drives spatio-temporal distribution of COVID-19 in china. *Nature* (to appear).

3. Aleta, A. *et al.* Modeling the impact of social distancing, testing, contact tracing and household quarantine on second-wave scenarios of the covid-19 epidemic. *medRxiv* (2020).

4. Lauer, S. A. *et al.* The incubation period of coronavirus disease 2019 (covid-19) from publicly reported confirmed cases: estimation and application. *Annals of internal medicine* **172**, 577–582 (2020).

5. Sohrabi, C. *et al.* World health organization declares global emergency: A review of the 2019 novel coronavirus (covid-19). *International Journal of Surgery* (2020).

6. Li, Q. *et al.* Early transmission dynamics in wuhan, china, of novel coronavirus–infected pneumonia. *New England Journal of Medicine* (2020).

7. Linton, N. M. *et al.* Incubation period and other epidemiological characteristics of 2019 novel coronavirus infections with right truncation: a statistical analysis of publicly available case data. *Journal of clinical medicine* **9**, 538 (2020).

8. Bi, Q. *et al.* Epidemiology and transmission of covid-19 in 391 cases and 1286 of their close contacts in shenzhen, china: a retrospective cohort study. *The Lancet Infectious Diseases* (2020).

9. Cao, M. *et al.* Clinical features of patients infected with the 2019 novel coronavirus (covid-19) in shanghai, china. *MedRxiv* (2020).

10. Chen, J. *et al.* Clinical progression of patients with covid-19 in shanghai, china. *Journal of Infection* (2020).

11. Cheng, Y. *et al.* Kidney disease is associated with in-hospital death of patients with covid-19. *Kidney international* (2020).

12. Clauset, A., Shalizi, C. R. & Newman, M. E. Power-law distributions in empirical data. *SIAM review* **51**, 661–703 (2009).

13. Centers for Disease Control and Prevention. Contact tracing for covid-19. `https://www.cdc.gov/coronavirus/2019-ncov/php/contact-tracing/contact-tracing-plan/contact-tracing` (2020).

14. Firth, J. A. *et al.* Combining fine-scale social contact data with epidemic modelling reveals interactions between contact tracing, quarantine, testing and physical distancing for controlling covid-19. *medRxiv* (2020).

15. Morley, J., Cowls, J., Taddeo, M. & Floridi, L. Ethical guidelines for covid-19 tracing apps (2020).

16. Gvili, Y. Security analysis of the covid-19 contact tracing specifications by apple inc. and google inc. *IACR Cryptol. ePrint Arch.* **2020**, 428 (2020).

17. Singapore Government. Tracetogether, safer together. `https://www.tracetogether.gov.sg/` (2020).

18. Australia Government Department of Health. Covidsafe app. `https://www.health.gov.au/resources/apps-and-tools/covidsafe-app` (2020).

19. National Cyber Security Centre. Nhs covid-19: the new contact-tracing app from the nhs. `https://www.ncsc.gov.uk/information/nhs-covid-19-app-explainer` (2020).

20. There are many reasons why COVID-19 contact-tracing apps may not work. Adam vaughan. `https://www.newscientist.com/article/2241041/` (2020).

21. Hinch, R. *et al.* Effective configurations of a digital contact tracing app: A report to nhsx. *en. In:(Apr. 2020). Available here. url: https://github. com/BDI-pathogens/covid-19_instant_tracing/blob/master/Report* (2020).
